# Quality by design approach identifies critical parameters driving oxygen delivery performance in vitro for perfluorocarbon based artificial oxygen carriers

**DOI:** 10.1038/s41598-021-84076-1

**Published:** 2021-03-10

**Authors:** Eric Lambert, Jelena M. Janjic

**Affiliations:** Graduate School of Pharmaceutical Sciences, Duquesne University, 600 Forbes Avenue, Pittsburgh, PA 15282 USA

**Keywords:** Nanomedicine, Nanoparticles, Biomaterials, Statistics

## Abstract

Perfluorocarbons (PFCs) exhibiting high solubility for oxygen are attractive materials as artificial oxygen carriers (AOC), an alternative to whole blood or Haemoglobin-based oxygen carriers (HBOCs). PFC-based AOCs, however, met clinical translation roadblocks due to product quality control challenges. To overcome these issues, we present an adaptation of Quality by Design (QbD) practices to optimization of PFC nanoemulsions (PFC-NEs) as AOCs. QbD elements including quality risk management, design of experiments (DoE), and multivariate data analysis facilitated the identification of composition and process parameters that strongly impacted PFC colloidal stability and oxygen transport function. Resulting quantitative relationships indicated a composition-driven tradeoff between stability and oxygen transport. It was found that PFC content was most predictive of in vitro oxygen release, but the PFC type (perfluoro-15-crown-5-ether, PCE or perfluorooctyl bromide, PFOB) had no effect on oxygen release. Furthermore, we found, under constant processing conditions, all PFC-NEs, comprised of varied PFC and hydrocarbon content, exhibited narrow droplet size range (100–150 nm) and narrow size distribution. Representative PFOB-NE maintained colloidal attributes upon manufacturing on larger scale (100 mL). QbD approach offers unique insights into PFC AOC performance, which will overcome current product development challenges and accelerate clinical translation.

## Introduction

Regenerative medicine and organ transplantation rely on successful organ preservation. Whole blood (WB) is a standard choice to restore oxygen in clinical settings, but it is far from a perfect product. For the patient, there are infectious risks, immune modulations, acute transfusion reactions, transfusion-related lung injury, volume overload, and hemolytic reactions^1^. Further, there are chemical and pharmacological drawbacks. First, there exist pH and temperature dependencies of oxygen dissociation from hemoglobin. This is problematic in conditions such as hypothermia, which is typically used in organ preservation. Second, hemoglobin relies on an allosteric effector, 2,3-diphosphoglycerate (2,3-DPG), to function properly. 2,3-DPG levels decrease with storage time which reduces performance^2^. Artificial oxygen carriers (AOC) have been developed with the goal of reducing need for WB^1^.

In the search for a versatile AOC, perfluorocarbons (PFCs) emerged as attractive materials because of their physicochemical properties that allow them to dissolve high amounts of gaseous species, including respiratory gasses^3^. In contrast to WB or blood products, PFC oxygen carrier function is minimally impacted by the environmental, chemical, and pharmacologic factors. Due to the nature of the association-dissociation mechanism of oxygen with PFCs that follows Henry’s Law linear dependence, PFCs as oxygen carriers are functionally resistant to pH and temperature influence. Due to the strength of the carbon–fluorine covalent bond, PFCs are chemically resistant to heat and do not undergo metabolic transformation in vivo. Therefore, PFCs are safer alternatives to Haemoglobin-based oxygen carriers (HBOCs), which can cause hypertension, immune reactions, and have short half-life^1,4^. Overview of AOC clinical translation challenges is discussed elsewhere^1,5^.

Injectable perfluorocarbon nanoemulsions (PFC-NEs) have adopted a range of uses such as blood transfusion substitutes^6^, support of cell growth in bioreactors ^7–10^, wound healing^11,12^, and in ischemia–reperfusion injury and tissue preservation^13–16^. However, clinical translation and approval of PFC-NEs are often met with challenges such as poorly-controlled colloidal stability^17^. Here we focus on the roadblocks of perfluorocarbon-based AOC development, which we hypothesize can be overcome by utilizing a systematic product development strategy, Quality by Design (QbD)^18^. Recently, we proposed QbD as a comprehensive approach to understanding colloidal attributes of PFC-NEs and their impact on performance across multiple biomedical applications, from imaging to oxygen delivery^18^. QbD has been applied to a variety of pharmaceutical systems ranging from powder blends to nanoemulsions^19,20^. Here, QbD is applied to PFC-NE colloidal stability and oxygen transport characterization, resulting in designation of formulation and processing design space for a model PFC AOC in which both attributes can be controlled.

QbD is a collection of practices that systematically facilitates pharmaceutical product development aimed at assuring product quality^20^. In QbD, critical quality attributes (CQAs) are defined as measurable product qualities that are crucial to the quality throughout the product lifetime. Quality risk management tabulates and ranks all ways in which CQAs can fail to meet CQA specifications (known as failure modes) leading to rational design of experiments (DoE). DoE facilitates efficient use of resources and eliminates the conventional resource-consuming one-factor-at-a-time approach to product optimization^21^.

In earlier notable PFC-AOC studies, authors derived mathematical equations to describe relationships between oxygen tension and distribution in blood vessels and tissue oxygenation under the influence of PFC emulsions^22,23^. More recently, empirical data was used to confirm finite element modeling findings showing that PFC-NE shelf-stability is impacted by PFC type and that oxygen diffusivity from PFC-NEs is impacted by interfacial area^24^. One study used response surface methodology to describe how droplet size and PFC loading efficiency were affected by processing parameters such as emulsification temperature, mixing time, and speed^25^. Others utilized an orthogonal design to evaluate the impact of composition on the particle size, polydispersity index (PDI), and zeta potential of perfluorooctyl bromide (PFOB) nanoemulsions^26^. However, none of these studies employed QbD. Here, we demonstrate the utility of QbD as opposed to one-factor-at-a-time (OFAT) approach.

Despite advances in preclinical models and computational studies, PFC-NEs have remained difficult to translate due to inadequate colloidal instability^27^. Enriching the dispersed PFC phase with a small amount of poorly water soluble hydrocarbon (HC) revealed a quick way to enhance stability^28^. However, the oils that opposed instability tended to have unacceptable organ residence times^27^. Alternatively, perfluorinated surfactants have been investigated to improve stability^3^. While they may be effective^29^, these structurally-novel materials have unknown toxicity and are thought to be environmental pollutants^30^. Therefore, creative and inventive solutions to development of PFC AOCs which provide rigorous understanding of colloidal attributes are in demand^18^. As an alternative to the above strategies, our group reported PFC-NEs with high colloidal stability by introducing hydrocarbon oil to the internal phase to form triphasic nanoemulsions, as opposed to biphasic, PFC in water systems^31–36^. These PFC-NEs achieved shelf-life beyond 360 days and were stable when mixed with biological media (no change after 14 days exposure).

Utilization of QbD elements has facilitated construction of predictive models for colloidal attributes^37^ and guided decisions in formulation and processing of triphasic nanoemulsion^38^. In this study, PFC-NEs were supplemented with medium chain triglycerides, a generally regarded as safe ingredient, to impart stability and a co-solubilizer to offer drug loading functionality in future efforts. Because the hydrocarbon shell component could restrict the oxygen release, both colloidal stability and in vitro oxygen transport ability were the primary focal attributes. A systematic product development approach was utilized: a detailed risk assessment (failure modes, effects, and criticality analysis, FMECA) identified key risks in formulation and processing. The risk assessment guided the construction of a DoE which sought to simultaneously evaluate colloidal stability and oxygen transport relationships, yielding meaningful product knowledge that screened for factors that impacted product attributes and identified a design space offering a visualization of the tradeoff existing between the two critical attributes. A representative nanoemulsion was manufactured at 4 × scale to demonstrate scalability.

## Results and discussion

Here, we present QbD-based development adapted to PFC-NE-based AOCs with two PFC molecules commonly reported from clinical studies: perfluoro-15-crown-5-ether (PCE^2,3^) and perfluorooctyl bromide (PFOB^13,39^.) These two molecules have been extensively used in regenerative medicine preclinical studies and have the most well established biodistribution kinetics in vivo^18^. The present PFC-NEs are formulated with medium chain triglycerides (MCT), Miglyol 812, to impart colloidal stability and a solubilizer, transcutol, to impart drug loading capacity for later use.

### Definition of product quality attributes and target specifications

As an essential preliminary-stage action and part of the QbD guidelines, product quality was defined by identifying CQAs and assigning acceptable specifications for each attribute. This step implemented measurable metrics to assess the degree of success of the oxygen carriers. CQAs were selected to reflect nanoemulsion colloidal stability and oxygen delivery performance (Table 1). The specifications or limits were chosen from a combination of prior knowledge and relevant literature reports^1,18,22,23,37,38^. For example, it is widely accepted that the larger a nanoparticle’s size, the more readily it is taken up by monocytes and macrophages. This results in rapid clearance from the body, which is undesirable in oxygen carriers. In summary, development of an oxygen carrier with both stability and oxygen loading has been an ongoing challenge; thus, defining product quality in this stage facilitated the development of a stable product proficient in oxygen transport. Definition of CQAs and target specifications makes up only part of the much broader quality target product profile within the QbD methodology, which is not within the scope of this report.

**Table 1:** Critical quality attributes (CQAs) for the reported perfluorocarbon nanoemulsions, specifications, and justifications.

CQA	Specification	Justification
Size (droplet diameter, nm)	100 < d < 200	Standard quality attribute in the field; necessary to understand the surface area available for release
PDI	< 0.2	Standard quality attribute in the field–indicative of stability
Zeta potential (mV)	− 25 ± 20	Standard quality attribute in the field–indicative of electrostatic repulsion
ΔSize after filtration	< 10% change from size measured before test	Sterilization required for parenterals
PDI after filtration	< 0.2	Sterilization required for parenterals
ΔSize after centrifugation	< 10% change from size measured before test	Cell culture demands centrifuging step
PDI after centrifugation	< 0.2	Cell culture demands centrifuging step
ΔSize after thermal storage at 50 °C	≤ 20% diameter change from day of production	Measure of accelerated stability
PDI after thermal storage at 50 °C	< 0.2	Measure of accelerated stability
ΔSize after 30 days storage at 4 °C	≤ 20% diameter change from day of production	Indication of shelf-stability
PDI after 30 days storage at 4 °C	< 0.2	Indication of shelf-stability
O_2_ loading and release	Dissolved oxygen maximum concentration in in vitro release, C_max_ ≥ 1.5 mg/L	Therapeutic evaluation

*PDI* polydispersity index, *d* diameter, *mV* millivolt, *nm* nanometer.

### Production, characterization, and quality control of PFC-NEs

Freshly prepared PFC-NEs were uniform, opaque liquids which ranged from 95 to 145 nm in diameter with polydispersity index < 0.2 and negative zeta potential. All formulations were periodically monitored for changes in size and size distribution for 30 days to measure colloidal stability during refrigeration. Additionally, samples were exposed to 50 °C for 2 weeks to simulate accelerated stability conditions, and size was recorded after exposure. The size change was converted to percent of initial size for analysis. This so-called thermal storage stability was of interest to verify stability was maintained during prolonged temperature rise. Ostwald ripening destabilization is governed by solubility, molar volume, and diffusion coefficient of the dispersed phase material^40,41^. All of these exhibit a temperature dependency that could accelerate mass transfer and droplet size growth. Following a detailed study reported in the literature of the colloidal behavior during autoclaving of triglyceride nanoemulsions stabilized by Poloxamers along with other reports of the prevalence of Ostwald ripening in PFC nanoemulsions^42^, it was expected that Ostwald ripening is the predominant mode of destabilization^43^. In the present study, three nanoemulsions exhibited size change beyond the specification for thermal storage. Samples were also sterile filtered through 0.22-μm-pore syringe filter and separate samples were centrifuged; in both cases samples retained droplet size and polydispersity. These two steps simulated sterilization and cell culture treatments, respectively. Table 2 provides all composition variables and processing conditions, and Table 3 tabulates CQA evaluations for all nanoemulsions. Supplemental Figures S1 and S2 give a graphical summary of the nanoemulsion characterization.

**Table 2:** List of formulations and the design matrix in 2-level, 6-factor d-optimal screening design of experiments.

Description (PFC concentration, vol%)	PFOB	PCE	Miglyol (MCT oil)	Transcutol	Micelle	Water	X1	X2	X3	X4	X5	X6
	Volume (mL)						Coded factor					
TN1 (2.8)	0.7	0	0.7	0.6	11.5	11.5	− 1	+ 1	− 1	+ 1	b	a
TN2 (5.0)	1.2495	0	1.2495	1.071	20.5275	0.9025	+ 1	+ 1	− 1	+ 1	b	a
TN3 (7.7)	0	1.9225	0.5765	1.071	20.5275	0.9025	+ 1	− 1	− 1	+ 1	a	b
TN4 (5.0)	0	1.2495	2.142	0.1785	20.5275	0.9025	+ 1	+ 1	− 1	− 1	a	b
TN5 (4.3)	0	1.0770	0.8230	0.1	11.5	11.5	− 1	− 1	− 1	− 1	a	a
TN6 (4.3)	1.0770	0	0.8230	0.1	11.5	11.5	− 1	− 1	− 1	− 1	b	b
TN7 (2.8)	0	0.7	0.7	0.6	11.5	11.5	− 1	+ 1	+ 1	+ 1	a	b
TN8 (7.7)	1.9225	0	1.4690	0.1785	20.5275	0.9025	+ 1	− 1	+ 1	− 1	b	a
BN1 (14.3)	3.57	0	0	0	20.5275	0.9025						
BN2 (14.3)	0	3.57	0	0	20.5275	0.9025						

The micelle solution was comprised of 2% w/v Pluronic P-105 and 3% w/v Pluronic P-123. X1, Internal phase fraction by vol [− 1 = 0.08, + 1 = 0.1428]; X2, hydrocarbon-to-perfluorocarbon (HC:PFC) ratio by volume [− 1 =− 0.857, + 1 = 1.857]; X3, Number of passes [− 1 = 4, + 1 = 6]; X4, Proportion transcutol in internal phase (IP) by vol [− 1 = 0.05, + 1 = 0.3]; X5, PFC type [a = perfluoro-15-crown-5-ether (PCE), b = perfluorooctyl bromide (PFOB)]; X6, Continuous media type [a = deionized water, b = normal saline]. BN, biphasic nanoemulsion; TN, triphasic nanoemulsion; MCT, medium chain triglyceride. Note: definitions and discussion of coded factors follows in the end of section Risk Management and Design of Experiments.

**Table 3:** Evaluation of critical quality attributes (CQAs). Cells with bolded font meet the CQA specifications.

NE code	CQA									
	Size (nm)	PDI	Zeta (mV)	Dissolved O_2_, C_max_ (mg/L)	Thermal stability ΔSize (%)	Thermal stability PDI	Filtration ΔSize (%)	Centrifugation ΔSize (%)	Day 30 ΔSize at 4 °C (%)	Day 30 PDI at 4 °C
BN1	**142.5**	**0.078**	**− 11.0**	**2.36**	151.7	**0.163**	**− 2.1**	**− 0.2**	125.8	**0.062**
BN2	**135.3**	**0.072**	**− 13.1**	**3.21**	26.3	**0.042**	**− 1.5**	**− 1.5**	**7.7**	**0.045**
TN1	**118.7**	**0.167**	**− 5.2**	1.16	**− 0.6**	**0.169**	**− 3.6**	**− 1.2**	**6.8**	**0.141**
TN2	**105.8**	**0.169**	**− 9.0**	**1.55**	**9.4**	**0.156**	**− 4.0**	**− 1.8**	**18.7**	**0.143**
TN3	**120.9**	**0.120**	− 3.1	**1.86**	24.0	**0.154**	**− 5.4**	**− 1.5**	**10.2**	**0.128**
TN4	**121.9**	**0.105**	− 1.9	1.46	**− 3.2**	**0.106**	**− 2.4**	**− 0.2**	**5.9**	**0.088**
TN5	**127.2**	**0.135**	**− 20.0**	1.32	**− 2.3**	**0.168**	**− 2.3**	**− 0.1**	**5.8**	**0.128**
TN6	**109.2**	**0.193**	− 3.8	1.31	**1.8**	**0.186**	**− 5.6**	**− 0.3**	**9.8**	**0.132**
TN7	**115.1**	**0.122**	− 2.9	1.27	**− 4.6**	**0.140**	**− 5.3**	**− 0.4**	**6.8**	**0.108**
TN8	98.1	**0.153**	**− 7.6**	**1.77**	**9.5**	**0.171**	**2.8**	**5.1**	**19.9**	**0.100**

* NE* nanoemulsion, *BN* biphasic nanoemulsion, *TN* triphasic nanoemulsion, *PDI* polydispersity index; C_max_, maximum dissolved O_2_ concentration.

### Evaluation of biphasic PFC-NEs as design controls to triphasic systems

**Figure 1:**
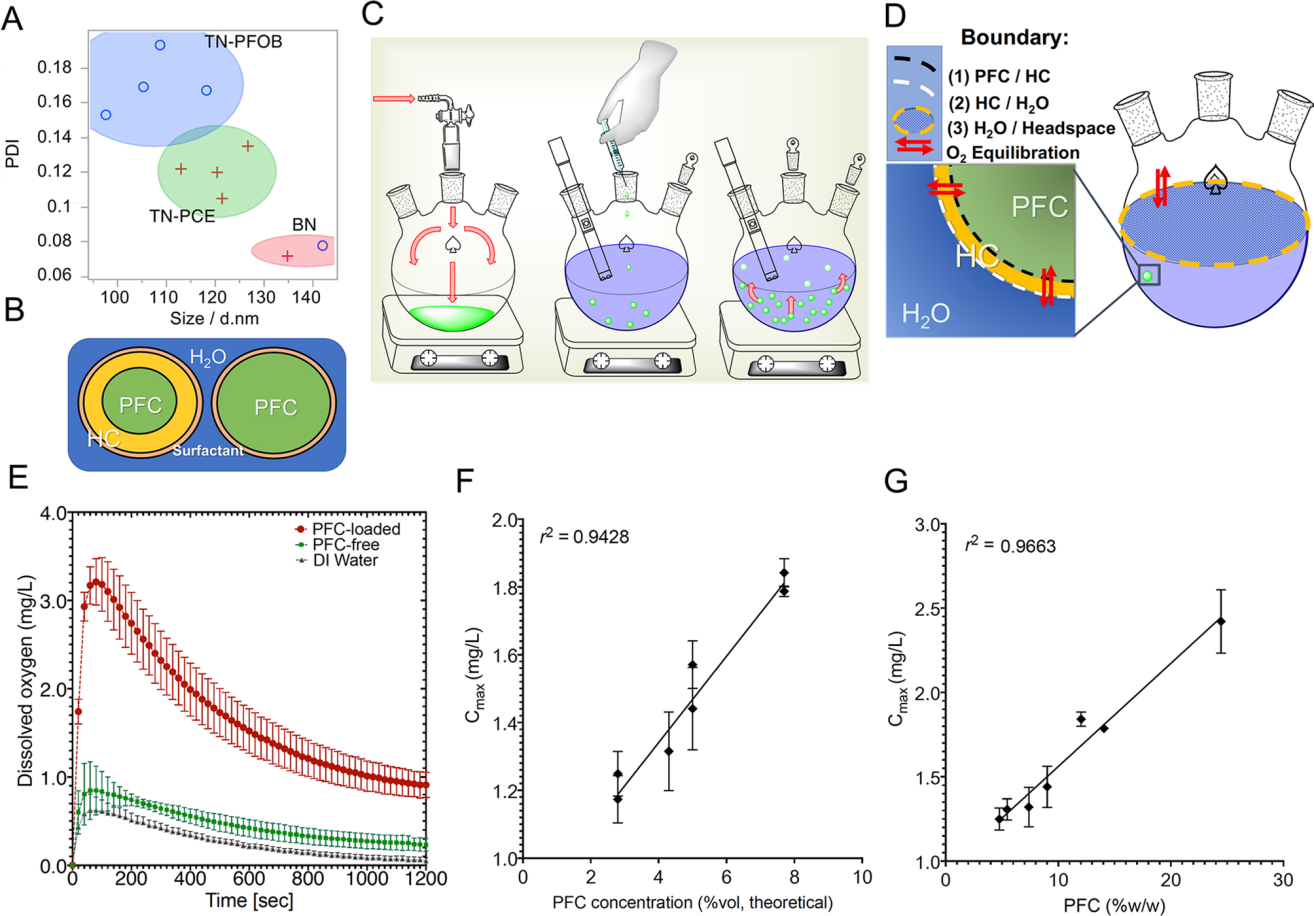
Summary of PFC nanoemulsions (PFC-NEs) for oxygen delivery. (**A**) Scatterplot of nanoemulsion z-average size and polydispersity index (PDI) of biphasic and triphasic nanoemulsions (BNs and TNs, respectively) consisting of perfluorooctyl bromide (PFOB) and perfluoro-15-crown-5-ether (PCE) (JMP Pro). (**B**) Illustration of the configurations of triphasic and biphasic nanoemulsions (TN and BN, respectively). (Microsoft PowerPoint) (**C**) Step-by-step in vitro oxygen loading and release experiments. The oxygen carrier sample is loaded by bubbling oxygen and then passing several passes of oxygen over the headspace. Meanwhile, release medium is deoxygenated by bubbling N_2_ and then passing several passes of N_2_ over the headspace. Oxygenated sample is removed by needle and syringe and injected into sealed release medium. Dissolved oxygen levels are monitored in the release medium (ChemDraw). (**D**) Schematic showing simplified sequence of interfaces that oxygen equilibrates across in the in vitro oxygen release setup. After loading the PFC-NE with O_2_, the majority of the dissolved oxygen resides in the PFC phase. There is no O_2_ in the release medium or the headspace of the round bottom flask. A concentration gradient drives the oxygen from the PFC core first into the hydrocarbon shell in the case of TN, then into the release medium, and finally into the headspace of the vessel. (ChemDraw) (**E**) Representative oxygen release data shows that PFC presence is essential for high levels of oxygen loading. (GraphPad Prism) (**F**, **G**) Dissolved O_2_ maximum concentration (C_max_) is linearly proportional to both theoretical PFC concentration, in percent volume, and PFC concentration quantified by ^19^F NMR spectroscopy, in percent weight PFC per weight of NMR sample (GraphPad Prism).

Next, we evaluated the oxygenation performance of biphasic nanoemulsions (BNs), (Fig. 1B, right configuration). We formulated biphasic PFC-NEs as comparison systems to triphasic nanoemulsions (TNs), (Fig. 1B, left configuration). Oxygen release measurements were conducted using an experimental setup illustrated in Fig. 1C. As oxygen partitioned from the NE droplets, it passed through the release medium, where dissolved oxygen levels were measured, and finally equilibrated into the headspace of the flask. Figure 1D details the compartments and boundaries across which oxygen equilibrated. The resulting oxygen release data, Fig. 1E, reflected the initial accumulation and subsequent dissipation of oxygen in the release medium. Full details appear in Materials and Methods. While this experimental setup demonstrated oxygen transport in a closed system well enough to allow us to differentiate between samples having different oxygen affinities, it is important to acknowledge that oxygen would be consumed by a living system and shear forces in the vasculature space would likely affect the convective diffusion.

Oxygen release was quantified and compared by extracting a maximum concentration from each dissolved oxygen release profile (C_max_), illustrated in Fig. 1E for biphasic nanoemulsions. This overlay compares a biphasic nanoemulsion comprised of 14.3%vol PCE (BN-PCE) to a PFC-free nanoemulsion (internal phase comprised of MCT at the same volume fraction) and to deionized water (containing no internal phase). Water as a control was important as it simulated the effect of using UW solution, the current gold standard in organ preservation^44^. The BN-PCE exhibited an increased oxygen transport activity compared to PFC-free NE and deionized water controls. The BN-PCE had a C_max_ of ~ 4 × and ~ 5 × those of PFC-free and water control. The 14.3%vol biphasic PFOB nanoemulsion (BN-PFOB) also demonstrated higher oxygen transport activity compared to the controls. These data support the potential of PFC-NEs in oxygen delivery.

Further evaluation of the oxygen transport activity revealed the linear nature of the oxygen affinity for PFC material. Figure 1F shows this relationship among all 8 TNs with respect to theoretical volume fraction of PFC. Expanding the search radius to include both BNs and TNs (TN3-TN8 and BN1, Fig. 1G) and employing quantified PFC concentration demonstrated the linear relationship was preserved. This suggested that the hydrocarbon shell had little effect on the oxygen release. Taking the oxygen release findings at face value suggested the biphasic PFC nanoemulsion was the optimal formulation. However, evaluation of the colloidal stability highlighted the risk associated with the biphasic formulation. Table 3 and Supplemental Figure S1 present key stability issues observed with biphasic formulations compared to triphasic nanoemulsions. Taking stability to be indicated by droplet size change and polydispersity changes over time and in response to accelerated conditions, it was readily noticed that BN-PFOB destabilized rapidly during shelf-storage conditions at 4 °C. BN-PCE maintained size through one month of storage, but did not endure the thermal storage test. Because both BNs failed to meet all colloidal stability CQA specifications, an alternative formulation was needed.

### Risk management and design of experiments

Previously collected data^37^ indicated that formulation modifications increased stability of PFC nanoemulsions. Lambert and Janjic^37^ demonstrated that inclusion of MCT shielded PFC-NEs from destabilization in serum- and salt-rich media. Other reports demonstrated similar claims, including supplementing with an insoluble oil decreases the Ostwald ripening rate^45,46^. This information provided guidance in our formulation development of stable PFC oxygen carriers, and we subsequently modified the composition to include an additional hydrocarbon component. These triphasic PFC nanoemulsions have been considered for a variety of applications (e.g. small molecule anti-inflammatory and imaging reagent carriers). Here triphasic nanoemulsions (TNs), comprised of a hydrocarbon phase (MCT and transcutol) and either PFOB or PCE, were investigated for their oxygen release performance in vitro.

**Figure 2:**
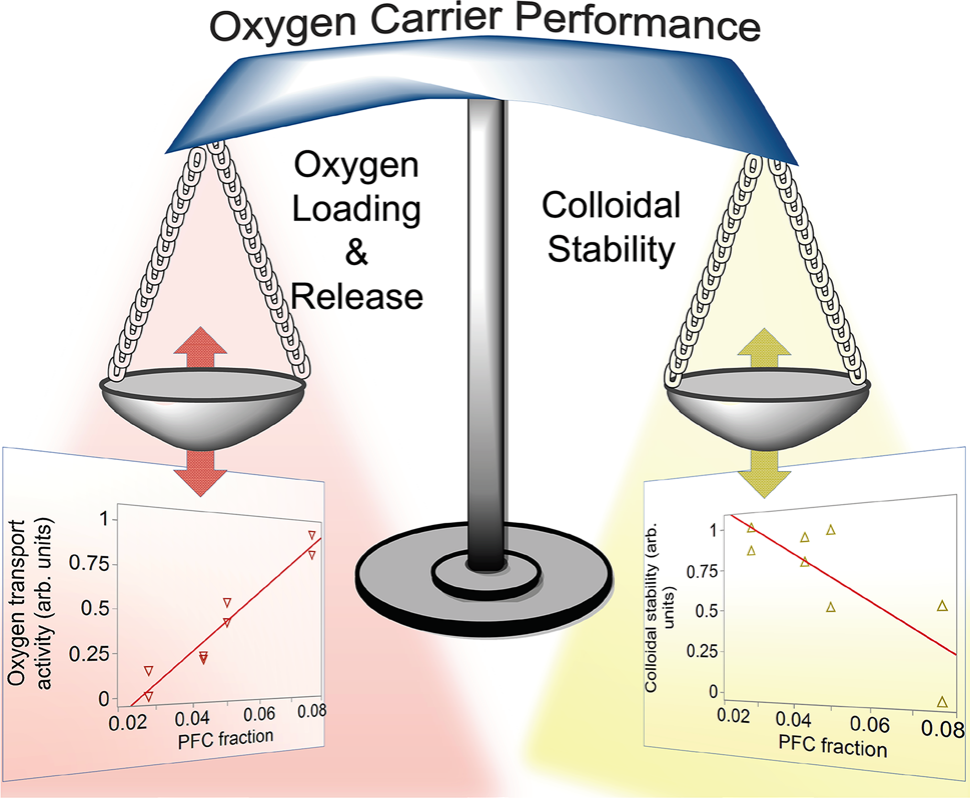
Balancing of opposing key attributes of PFC-NE O_2_ carriers. Highly concentrated PFC-NEs are sought for their high capacity for oxygen loading. However, biphasic PFC-NEs can be poorly stable. A key objective is to find a balance between these opposing quality attributes. Conceptual scatterplots (formed using JMP Pro) were formed by normalizing CQAs C_max_ and Thermal stability Δsize into a number ranging from 0 to 1 by dividing each by the maximum respective CQA measurement (Balance graphic drawn in ChemDraw).

It was anticipated that adding a hydrocarbon oil could compromise the activity of the oxygen-rich PFC phase, so subsequent product development applied concepts of QbD to investigate the (i) colloidal stability and (ii) oxygen transport activity of this triphasic configuration (PFC-in-HC-in-water). The schematic in Fig. 2 illustrates the conceptual balance of these two opposing attributes. Data throughout the text reinforce that understanding and controlling this balance is crucial to the development of an AOC. To provide direction and structure within this formulation space in this stage of product development, risk management followed by statistical design of experiments played a central role in evaluating triphasic nanoemulsions.

**Figure 3:**
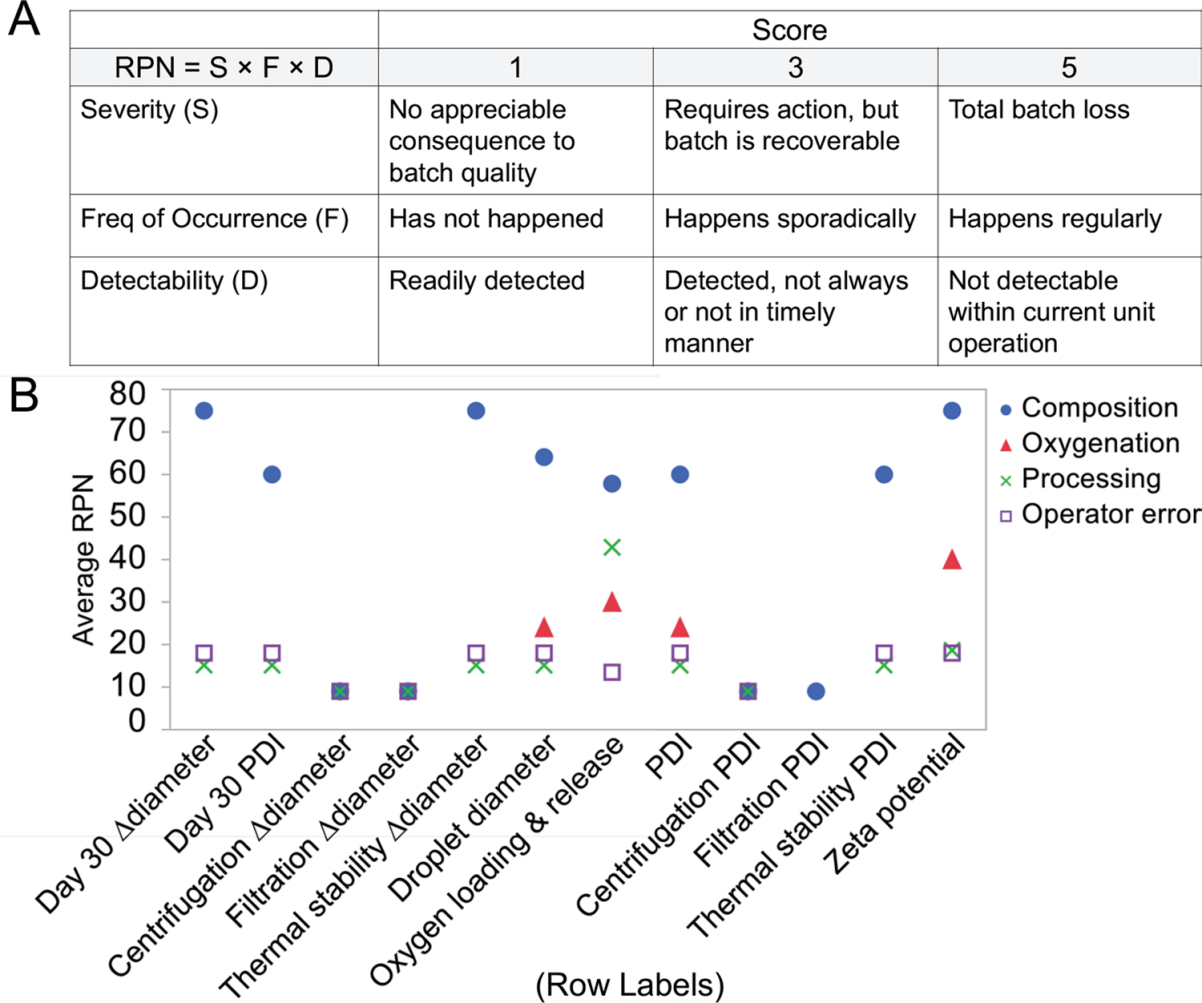
Description and summary of failure mode, effects, and criticality analysis (FMECA) results. (**A**) Definition of Risk Priority Number (RPN) scoring system. Each failure mode in a specified critical quality attribute is assigned scores in each of three categories (Severity, Frequency of Occurrence, and Detectability). RPN is calculated by the product of these individual scores. Scores of 2 and 4 would indicate the failure mode is between 1 and 3, and between 3 and 5, respectively (Microsoft PowerPoint). (**B**) For each critical quality attribute, all failure modes were recorded, and each was classified into one of 4 broad failure modes category (composition, oxygenation, processing, or operator error). All RPN from each failure mode for each CQA in a specified category were averaged to highlight which of the 4 broad failure modes categories were most likely to present challenges in PFC-NE development (JMP Pro).

A risk management strategy called failure mode, effects, and criticality analysis (FMECA) examined and ranked potential sources and mechanisms of CQA specification failure. Full details on FMECA risk calculations appear in Materials and Methods. This methodology identified that composition-related factors were the most critical failure modes. Figure 3 summarizes the initial risk assessment. Here, the average risk priority number (RPN) for all failure modes is collected for each CQA, highlighting the risk associated with the collective average of entries for each of four broad failure modes categories (composition, oxygenation, processing, or operator error). In the unabridged FMECA, each entry is broken down further by the specific failure mode causes. For example, inappropriate hydrocarbon: perfluorocarbon (HC: PFC) ratio (belonging to the broad failure mode inappropriate composition) causes oxygen release to fall out of specification because the hydrocarbon shell limits the oxygen transfer rate at high ratios.. To appreciate a more comprehensive look at the failure modes, cause and effect diagrams are shown in Supplemental Figure S6 for Thermal stability Δsize and C_max_ in which sources of variability for both responses appear in expanded detail. FMECA results directly informed which factors appeared in the experimental design. Because FMECA assigned highest risk to composition-related factors, a d-optimal design of experiments selected five composition factors and one processing factor to study. Design factors and levels are provided in Table 2.

In this screening experimental design, PFC-NE composition was represented by five factors: PFC type, continuous media type, internal phase (IP) volume fraction, hydrocarbon: perfluorocarbon ratio by volume (HC: PFC ratio), and proportion transcutol in IP by volume. Three of these: IP volume fraction (X1), HC: PFC ratio (X2), and proportion transcutol (X4) reflected the absolute and relative concentrations of IP components. X1 was the volume of internal phase components divided by the total NE volume. This was a measure of emulsion droplet concentration. In all cases, surfactant amount was adjusted such that all emulsion formulations had identical surfactant to oil ratios.

X2 was a unitless quantity indicative of relative volumes of PFC (core compartment) and HC (shell compartment) components in the internal phase. The higher the ratio, the lower PFC amount in the formulation. This also meant the hydrocarbon shell surrounding the PFC phase was thicker, which possibly imparted resistance to oxygen release. X4 was the volume fraction of the internal phase that was comprised of transcutol, a versatile co-solubilizer. Our group previously used transcutol to increase loading of celecoxib by 10 times the dose of a nanoemulsion delivery platform which had effect treating rat chronic pain model^38,47^. When adjusting X4, it was added at the expense of the MCT oil. These three were chosen as they collectively reflect the concentration of internal phase components and could be varied in DoE to reduce multicollinearity in statistical regression analysis. PFC type (X5) and continuous medium (X6) were categorical formulation factors. Perfluorooctyl bromide (PFOB) and perfluoro-15-crown-5-ether (PCE) are nonvolatile perfluorocarbon oils (boiling points are 142 and 145 °C, respectively). Number of passes (X3) was the single processing parameter chosen to study based on our prior work^18,37,38^. It was anticipated that nanoemulsion droplet size would impact the oxygen release profile by affecting the surface area available for oxygen exchange. Adjusting the number of re-circulations on the microfluidizer was a direct way to manipulate droplet size while keeping composition and concentration constant. This 6-factor, 2-level DoE was suited to screen and identify factors active in impacting attributes.

### Evaluation of Triphasic PFC nanoemulsions

Table 3 shows that triphasic nanoemulsions (TNs) exhibited a small but consistent size reduction and increase in size distribution compared to biphasic nanoemulsions (BNs). Further, a discrepancy in size and PDI was seen between PFC types. This is highlighted in a scatterplot of size vs. PDI and cluster analysis which defined three distinct clusters (Fig. 1A). Zeta potential remained negative, but four TNs exhibited zeta magnitude lower than the CQA specification. Even so, it was evident that TNs exhibited improved colloidal stability compared to the BNs. All triphasic NEs maintained size in both centrifuge and filtration test. Only one TN failed to meet thermal stability Δsize specification and none failed to meet the day 30 Δsize specifications. TN3, the triphasic formulation that failed to meet thermal stability spec, consisted of the lowest amount of MCT. These observations suggested that electrostatic repulsion was not the principal mode of stabilization and that MCT acted as a stability enhancer.

**Figure 4:**
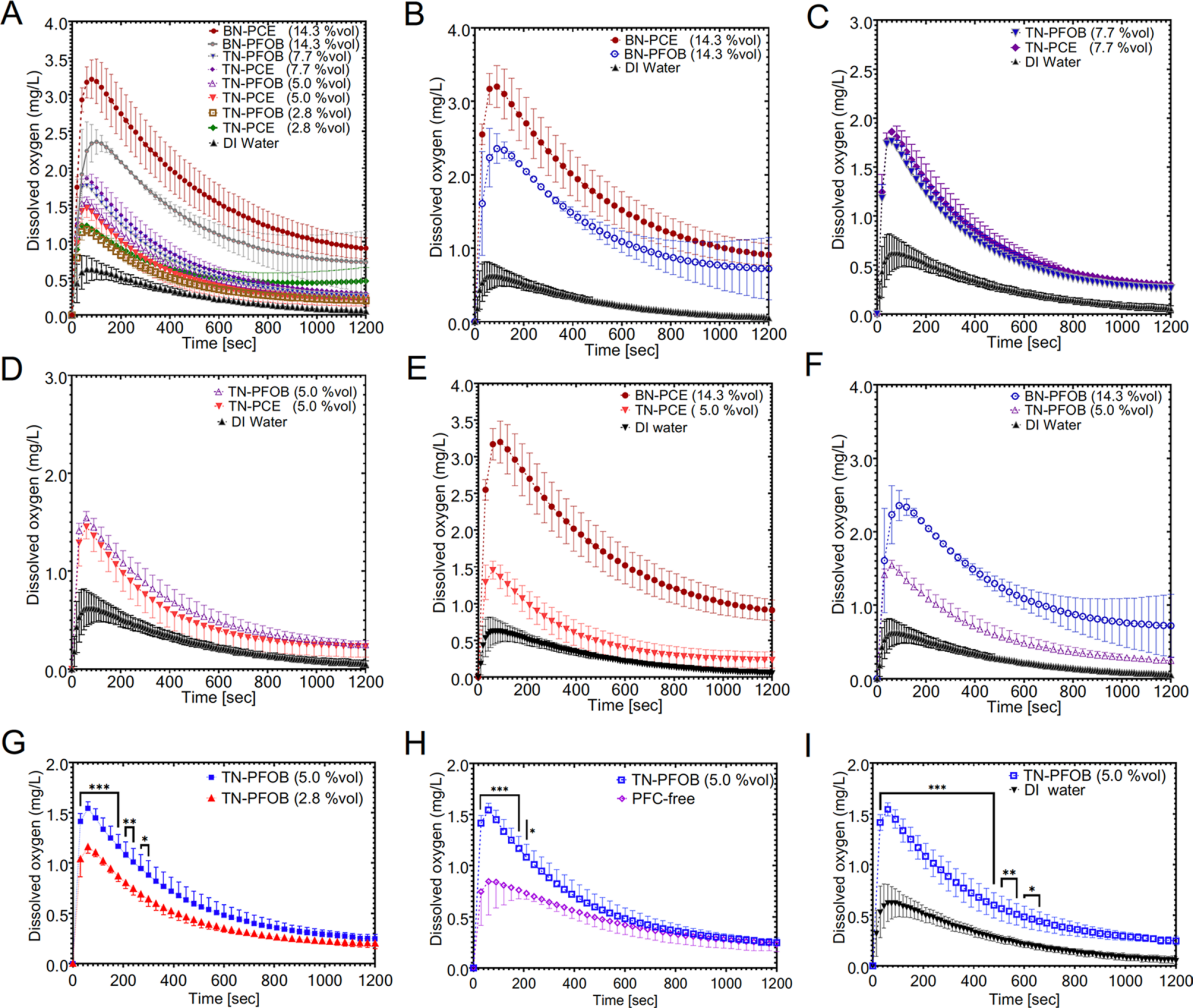
Dissolved oxygen vs. time profiles in in vitro oxygen release experiments for select samples. In each release profile, a maximum dissolved oxygen concentration (C_max_) was extracted and used as the metric by which different samples were compared. (**A**) Release profiles compiled for formulations ranging from 0 to 14.3 vol% PFC. (**B**) Comparison of BNs composed of different PFC types, both at the highest level of PFC. (**C**, **D**) Comparison of 2 TNs composed of different PFC types at identical PFC levels. (**E**, **F**) comparison of BNs and TNs of the same PFC type. (**G**) Comparison of TN-PFOB of different internal phase volume fractions. (**H**, **I**) Comparison of TN-PFOB to biphasic hydrocarbon nanoemulsion and DI water control. Where standard error bars are shown, each point is mean of three measurements ± SD. Asterisks label time points or intervals where the two overlaid dissolved oxygen values are significantly different from one another. *, **, *** indicate *P* < 0.05, *P* < 0.01, and *P* < 0.001, respectively. Graphs and analysis done in GraphPad Prism.

**Figure 5:**
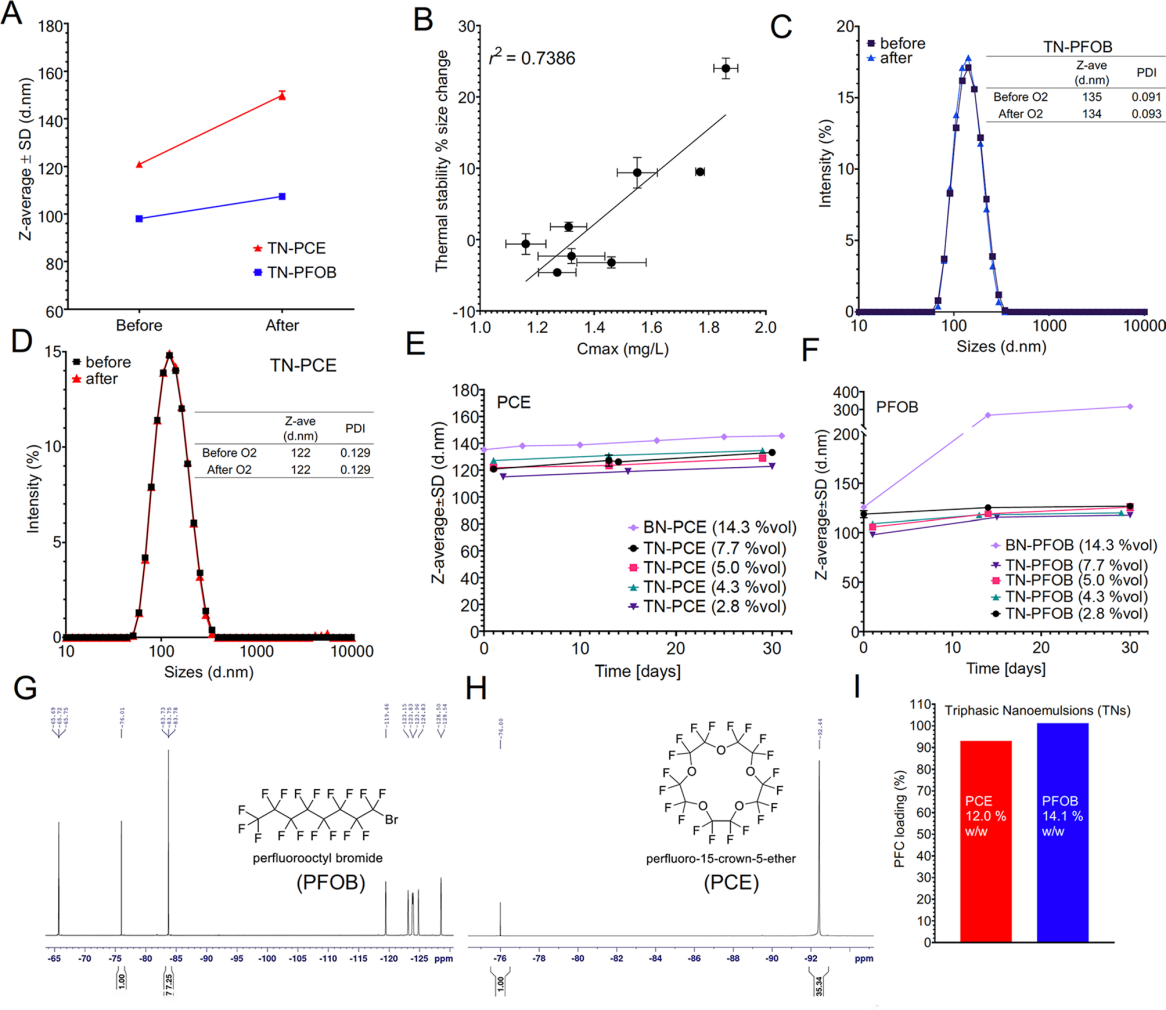
Characterization of triphasic PFC nanoemulsions. (**A**) For TNs, oxygen release is virtually identical among PFC types comparison in (see **Fig. ****4**). Thermal stability Δsize for 2 representative samples shows a colloidal stability discrepancy exists between PFC types. (**B**) Least squares fit demonstrating the relationship of C_max_ and Thermal stability Δsize. (**C**, **D**) Size distribution overlays reflect colloidal stability of TNs after 30 min of oxygen bubbling. (**E**, **F**) Shelf-stability reflected by z-average of samples stored at 4 °C. (**G**, **H**) ^19^F NMR spectra of representative examples, TN3 and TN8 (TopSpin). Chemical drawings were done in ChemDraw. (**I**) PFC loading calculated from ^19^F qNMR spectroscopy. Graphs were made in GraphPad Prism.

After observing the corresponding shift in colloidal stability with the introduction of MCT oil, oxygen transport activity of TNs was assessed. Presented in Fig. 4 are notable dissolved oxygen concentration–time comparisons. Maximum concentration (C_max_) was attained on the order of one minute into the release for all samples. The C_max_ numbers observed in this experiment matched those reported by others^50,51^ in similar experimental setups. Panel A compares three TNs consisting of different levels of PFC concentration for each PFC type along with the BNs. The C_max_ increased with increasing PFC amount, and all PFC-NEs had higher C_max_ than water. Figure 4B-D each compare PFC type. Figure 4B contrasts PFC type for BNs. There was a difference in C_max_ for PFC type for BNs. Figure 4C displays TN samples with different PFC types for 7.7%vol and Fig. 4D displays TN samples with different PFC types for 5%vol. PFC type had no detectable effect on the oxygen release of TNs. This apparent contradiction may be explained by considering the poor colloidal stability of BNs . Droplet size of BN-PFOB rapidly grew in storage, whereas BN-PCE maintained size for 1 month (see Fig. 5E-F as well as Supplemental Figure S3.). This size increase would decrease the surface area available for release and could account for the apparent difference in C_max_ between PFC type in the BN comparison. Figure 4E-F compare samples of like PFC and like volume fractions, but different PFC concentrations. In each case, the sample with higher PFC concentration exhibited higher C_max_. This supports the idea that the hydrocarbon shell dissolved less oxygen and may have obstructed the release of oxygen from the PFC core. Figure 4G compares TNs comprised of different volume fractions. The oxygen release was significantly greater for the sample with higher volume fraction for nearly six minutes of the release and remained, on average, higher for the duration of the release. This effect was attributed to the higher amount of material in the NE. This trend is supported by Fig. 1F-G. Although the TN exhibiting the higher C_max_ contained 1.8 × higher PFC concentration (5.0 vs. 2.8%vol), the C_max_ was less than 1.4 × higher (1.546 vs. 1.162 mg/L) than the TN consisting of less PFC. This was because the aqueous continuous phase and hydrocarbon compartment of the internal phase were saturated with oxygen and contributed to the release. Figure 4H shows the TN-PFOB (5% vol) compared to HC biphasic nanoemulsion (PFC-free). These two NEs had the same volume fraction, and thus made a clear comparison between PFC and HC material. Figure 4I compares TN-PFOB (5% vol) to water. These comparisons showed a higher C_max_ for the TN-PFOB and confirmed that PFC material was essential for oxygen transport. Overall, the oxygen release was highly affected by PFC content. This was observed in NEs containing higher overall volume fraction (Fig. 4G), and in NEs comprised of equal volume fractions but differing in concentration of PFC (Fig. 4H). It is worth noting that exchanging perfluorocarbon type had negligible effect, suggested by Fig. 4C-D the linear nature of the C_max_—PFC loading relationship (Fig. 1F-G). Further, the presence of salt in the continuous media did not impact the oxygen release profile.

Evaluation of triphasic nanoemulsions revealed virtually identical oxygen release profile between PFC type (Fig. 4C-D) and slight discrepancies in thermal stability Δsize between PFC type (Fig. 5A) that appeared to be a result of low MCT amount. Additionally, it was observed that C_max_ and thermal stability Δsize were positively correlated (Fig. 5B). This finding represented the key challenge and was central in supporting other reports of PFC oxygen carriers. Recognizing the stability differences between PFC types, we further evaluated colloidal stability of triphasic PFC-NEs. While bubbling gas through a nanoemulsion was a simple way to perform oxygen loading, we wanted to ensure this step would not mechanically disturb the emulsion and lead to destabilization. Figure 5C-D are representative size distribution measurements taken before and after the oxygen bubbling step. It was observed that both TNs maintained size distribution. While no significant difference between PFC types in C_max_ was observed, we wanted to verify the PFC loading was not different. Figure 5I shows representative ^19^F NMR quantification, suggesting PFC loading in triphasics was similar for two PFC types. NMR spectra corresponding to Fig. 5I are shown in Fig. 5G-H.

### Regression model construction and evaluation

Multiple linear regression (MLR) was utilized to fit models that described C_max_ and Thermal stability Δsize in terms of the DoE factors. Reduced MLR models for both C_max_ and Thermal stability Δsize appear in Table 4.

**Table 4:** Reduced regression model terms and goodness-of-fit for C_max_ and thermal stability Δsize.

Response	Model term	Model term estimate	Model term SE	t Ratio	*P*-value	Model *R*^2^	Model RMSE
C_max_	Intercept	1.480	0.0110	134.32	< .0001	0.995	0.027 (mg/L)
	X1 (Internal phase fraction)	0.198	0.0095	20.78	0.0002		
	X2 (HC:PFC ratio)	− 0.136	0.0110	− 12.30	0.0012		
	X3 (Number of passes)	0.038	0.0110	3.41	0.0422		
	X4 (Proportion transcutol in IP)	0.065	0.0110	5.89	0.0098		
Thermal stability ΔSize	Intercept	4.250	0.7705	5.52	0.0053	0.971	2.179 (%)
	X1 (Internal phase fraction)	5.675	0.7705	7.37	0.0018		
	X2 (HC:PFC ratio)	− 7.200	0.8896	− 8.09	0.0013		
	X4 (Proportion transcutol in IP)	6.400	0.8896	7.19	0.0020		

*HC* hydrocarbon, *PFC* perfluorocarbon, *IP* internal phase, *R*^2^ Coefficient of Determination,*RMSE* root mean square error.

The terms X1, X2, X3, and X4 were present in the reduced C_max_ model. While the oxygen solubility governed how much oxygen can be loaded and released, PFOB and PCE had similar enough oxygen affinities to not see a significant difference between their oxygen transport activities. This was first suggested by data in Fig. 4C and corroborated by elimination of X5 from the model. Similarly, X6 was removed during regression analysis. While salinity affects gas solubility in water, the main carrier of oxygen in the nanoemulsion was the PFC phase. Therefore, the reduction in oxygen loading in the aqueous phase when adjusting for tonicity resulted in negligible oxygen-carrying loss. Estimates for X1 and X2, terms which represented the amount of PFC, had positive and negative signs, respectively. Consistent with data shared in previous sections, higher C_max_ resulted from pairing higher internal phase fraction (positive X1) with lower HC:PFC ratio (negative X2), ultimately yielding more PFC content. Further, C_max_ was dependent on X3. More passes resulted in more surface area available for oxygen transfer.

For the reduced thermal stability Δsize model, X1, X2, and X4 appeared as model terms. X2 and X4 collectively determined the amount of MCT. As anticipated, high X2, where MCT oil is high, suppressed size growth and high X4, where MCT oil is reduced, promoted size growth. Both models had these three terms in common, but they had opposing effects in each response’s model, supporting data in Fig. 5B.

**Figure 6:**
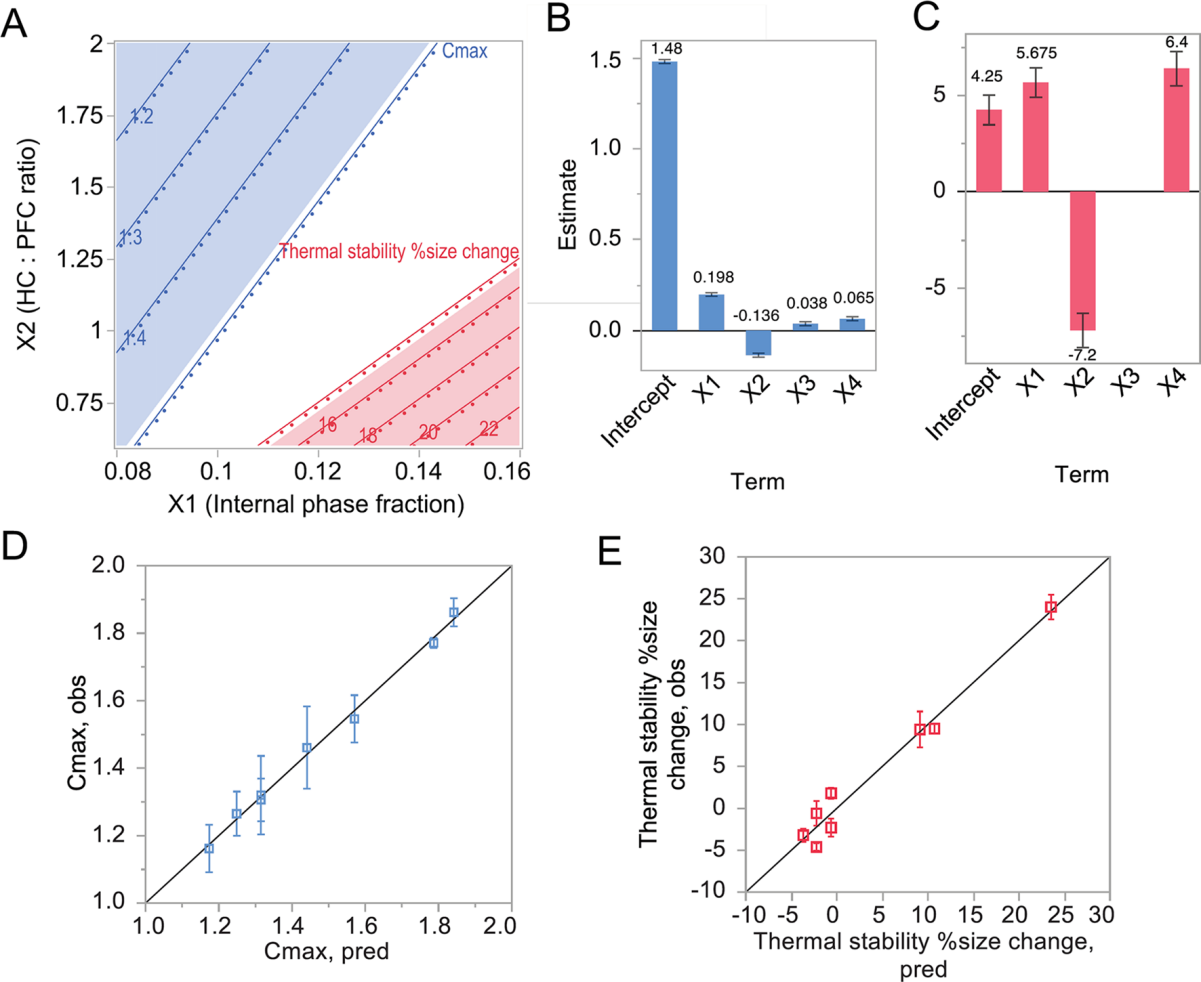
Multiple linear regression output to predict size change after thermal storage and C_max_ in oxygen release. (**A**) Contour plot representing the formulation space in X1 and X2 identified by regression models for both responses. Shaded regions are out-of-specification regions for C_max_ (blue) and Thermal stability Δsize (red). (**B**-**C**) Regression term estimates for C_max_ (blue) and Thermal stability Δsize (red). (**D**-**E**) Observed by predicted graphs visualize the goodness of fit for both models, where C_max_ is blue squares and Thermal stability Δsize is red squares. Graphs and analysis done in JMP Pro.

The formulation space described by regression models for C_max_ and Thermal stability Δsize is summarized in Fig. 6 along with regression term estimates (B-C) and actual by predicted plots (D-E). Panel A shows a plot overlaying contours of both responses in formulation space X1 and X2. The shaded blue and red regions represent where C_max_ and Thermal stability Δsize, respectively, were outside of specifications, highlighting this opposing nature of the two responses. Model goodness-of-fit was reflected by the appearance of the data in observed by predicted plots falling along the diagonal 1:1 line in Panels D and E.

In the triphasic formulations, the makeup of the dispersed phase (i.e. volume fraction, proportion transcutol, HC: PFC ratio) was the most critical feature for both responses. C_max_ and thermal stability were both described by these factors, but with opposing outcomes. Importantly, the DoE regression analysis provided quantitative relationships describing both responses. This allowed simultaneous monitoring of both CQAs during product development to achieve a PFC nanoemulsion formulation that satisfied both CQA specifications while minimizing material and time resources. In addition, analysis highlighted X3 impacted C_max_ but not Thermal stability Δsize. This key finding indicated processing could be tuned to enhance oxygen transport without affecting colloidal stability.

### Scale-up of PFOB nanoemulsions

**Figure 7:**
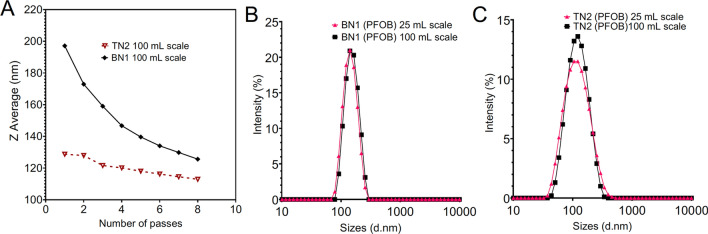
Characterization and scale-up of PFC nanoemulsions. (**A**) Dependence of 100 mL NE size on processing amount (n = 1 measurement/ data point). (**B**) Size distribution overlays of biphasic PFOB, BN1, at 25- and 100-mL scale. (**C**) Size distribution overlays of triphasic PFOB, TN2, at 25- and 100-mL scale. Graphs from GraphPad Prism.

To demonstrate scalability, one of the more PFC-rich TNs (TN2) and a BN of like PFC type (BN1) were chosen to be produced at 4 × scale (100 mL). Figure 7B-C shows that the scaled-up emulsions maintained monomodal size distribution. Because producing nanoemulsions in larger batches is essential for moving into pre-clinical and clinical phases of development, this finding was encouraging. Figure 7A shows the dependence of size on number of passes for 100-mL NEs. The biphasic formulation had a steeper dependence on processing time compared to the triphasic. This indicates the triphasic NE was produced at a specified size with less processing than the biphasic NE, which was important to acknowledge alongside the finding that more recirculations improves C_max_ without affecting Thermal stability Δsize.

## Conclusions

We have presented an implementation of systematic pharmaceutical product development methodology, QbD, to formulation of PFC-NEs as AOCs. Through this process, we identified critical quality attributes that reflected colloidal stability and oxygen delivery performance. Furthermore, quality risk management was implemented to enable rational formulation development where both composition and processing parameters were evaluated. The risk assessment guided creation of DoE used to construct regression models which described two competing quality attributes, oxygen release and colloidal stability. We found that the triphasic NE colloidal stability exceeded colloidal stability of biphasic NE, while the biphasic NEs demonstrated highest oxygen loading. Further, increase in PFC loading led to increase in oxygen loading. We showed that presented nanoemulsions remained stable under oxygen exposure in vitro (Fig. 5C-D). We also established scalable processing parameters which was deemed successful by producing 100 mL-scale nanoemulsions.

This study in its current form presents certain limitations. First, only two commonly used perfluorocarbons were used. Second, all oxygen release studies were performed in vitro under very controlled conditions. Future studies will expand to assessing presented PFC-NEs in organ preservation testing. Due to small sample size, we were not able to fully quantify the impact of hydrocarbon layer on the oxygen loading. Our data suggested that regardless of hydrocarbon presence, the loading capacity of presented nanoemulsions was directly linearly correlated to PFC content levels.

## Materials and methods

### Materials

Pluronic P-105 was purchased from BASF (Florham Park, New Jersey, USA). Pluronic P-123 was purchased from Millipore Sigma (St. Louis, Missouri). Perfluorooctyl bromide and perfluoro-15-crown-5-ether were purchased from Exfluor Research Corp (Round Rock, Texas, USA). Miglyol 812 (capric acid/caprylic acid triglycerides, known as medium chain triglycerides, MCT) was obtained from CREMER Oleo (Hamburg, Germany). Transcutol was purchased from Spectrum Chemical Mfg Corp (New Brunswick, New Jersey, USA). Trifluoroacetic acid and deuterium oxide, 99.8% were obtained from Acros Organics (Thermo Fisher Scientific, Waltham, Massachusetts, U.S.). Dulbecco’s Modified Eagle’s Medium was supplied by Corning (Corning, New York, U.S.), and fetal bovine serum was supplied by ATCC (Manassas, Virginia). All chemicals and reagents were used without modification.

### Failure mode, effects, and criticality analysis

Formulation and manufacturing of nanoemulsions was examined one unit operation at a time. In each unit operation, all sources of variability of critical quality attributes were identified and treated as a failure mode. All failure modes in all critical quality attributes were evaluated by assigning a Risk Priority Number (RPN), which was calculated as^52,53^:

$$RPN = Severity \times Frequency\;of\;Occurrence \times Detectability$$

Severity, frequency of occurrence, and detectability associated with a failure mode were scored on a scale of 1–5, low to high risk. Specifically, ‘no appreciable consequence to batch quality’, ‘has not happened’, and ‘readily detected’ corresponded to a score of 1 for severity, frequency of occurrence, and detectability, respectively. ‘Total batch loss’, ‘happens regularly’, and ‘not detectable within current unit operation’ corresponded to a score of 5 for severity, frequency of occurrence, and detectability, respectively. Accordingly, the higher the RPN, the more risk associated with the corresponding failure mode.

### Design of experiments

Five composition factors and one processing factor were each varied at two levels in a D-optimal screening design^54^ of experiments (DoE) consisting of 8 experimental runs created using JMP Pro 14.3.0 (SAS, Cary, NC, USA). In this design, the impact of all 6 main effects on emulsion attributes was statistically evaluated. Composition factors included PFC type, continuous media type, internal phase (IP) volume fraction, hydrocarbon-to-perfluorocarbon ratio by volume (HC: PFC ratio), and proportion transcutol in IP by volume. The lone processing factor was number of recirculations (passes) through microfluidization interaction chamber. Table 2 compiles all experimental runs in the DoE. Emulsion attributes included droplet diameter, polydispersity, zeta potential, resistance to size change (i) under storage at 4 °C, (ii) under exposure to centrifugation, (iii) under filtration, (iv) and under storage at elevated temperature, and oxygen release profile (see Colloidal Characterization in Methods section).

### Data processing and regression model construction

Regression models were constructed using multiple linear regression. Using backwards stepwise variable selection and selecting *P* value < 0.05 as the significance threshold, model terms with *P* > 0.05 were removed one at a time to form reduced regression models. Coefficient of determination (*R*^2^), root mean square error (RMSE), and leave-one-out cross validation (LOOCV) were used to assess model goodness-of-fit. ANOVA and model fit statistics are given in full detail in Supplemental Tables S1-S2.

### Production of triphasic nanoemulsions

All nanoemulsions were produced following earlier published protocols^18,33,36,38^. Briefly, a micelle solution of blended non-ionic surfactants (2% Pluronic P-105, 3% Pluronic P-123 w/v) was made by following previously reported protocol with minor modifications^55^. To prepare nanoemulsions, micelle solution was added to the mixture of PFC and HC oils, or single (PFC or HC) oil. Then, coarse pre-emulsions were produced with an analog vortex mixer (VWR, Radnor, PA, USA) on high for 30 s. Coarse emulsions were sonicated on ice for 30 s at 29% amplitude (equivalent of 3480 W s) with Model 450 Digital Sonifier (BRANSON Ultrasonics Corporation, Danbury, CT, USA). Microfluidization was performed on all coarse emulsions by Microfluidizer M110S (Microfluidics Corp., Westwood, MA, USA) at 15,000 psi liquid pressure for the specified number of pulses (5 pulses to 1 pass) over an ice-cold interaction chamber. Emulsions were packaged in glass vials and stored at 4 °C.

### Colloidal characterization and quality control

Fresh nanoemulsion samples were diluted 1:40 v/v in DI water and size distribution (described by a z-average droplet diameter and polydispersity index (PDI)) and zeta potential were measured by dynamic light scattering (DLS) on Zetasizer Nano (Malvern Instruments, Worcestershire, UK). DLS operating parameters were as follows: refractive indices of material, 1.59, and dispersant 1.33; viscosity of the dispersant, 0.8872 cP; temperature, 25 °C; duration of measurements, automatically determined by the number of runs, with each run 10 s; and 173 degrees backscatter angle. Size distribution and zeta potential of refrigerated samples were monitored periodically over time as a measure of shelf stability. Alternative measures of colloidal stability included centrifugation, filtration, serum incubation stability, and thermal stability, in which size was measured before and after each stability test. Percent size change relative to the size before stress was recorded. For the centrifugation test, sample was diluted 1:40 v/v in DI water and centrifuged (Centrifuge 5804 R, VWR, Eppendorf AG, Hamburg, Germany). at 1100 RPM (16.1 × *g*) for 5 min at room temperature. For the filtration test, sample was filtered through 0.22 μm-pore mixed cellulose ester syringe filter. For the serum test, sample was diluted 1:40 v/v in DI water, Dulbecco’s Modified Eagle’s Medium (DMEM), or 20% fetal bovine serum in DMEM. Sample was stored for 72 h at 37 °C. For the thermal stability test, sample was aliquoted and stored at 50 °C for two weeks. All colloidal stability tests were begun on samples 1 week or less after preparation. Supplemental Figures S1 – S5 show graphically the results of all tests. Individual unpaired, two-tail t-tests were done on the quality control tests and are annotated on the bar graphs in Supplemental Figures S1 and S2. DLS data were summarized by mean ± standard deviation of 3 measurements unless otherwise specified.

### Oxygen release kinetics

2.5 mL of sample (AOC or control) was dispensed into a 100-mL 3-necked round bottom flask under magnetic stirring. Oxygen gas was bubbled into sample through glass Pasteur pipette for 30 min. Oxygen gas was then passed over the sample to fill the headspace volume with pure oxygen and the flask was sealed from the surrounding environment with rubber stoppers. Meanwhile, deionized water (25 mL) in a 100-mL 3-necked round bottom flask under magnetic stirring as the release medium was degassed with nitrogen gas until the submerged dissolved oxygen probe (HI5421 Dissolved Oxygen Benchtop meter Hanna Instruments, Woonsocket, Rhode Island, USA) gave a stable reading of zero oxygen in the aqueous release medium. Nitrogen gas was passed over the water to remove any residual oxygen in the headspace, and the flask was sealed from the surroundings with rubber stoppers. Oxygenated sample (1 mL) was removed with syringe and needle and injected into flask containing degassed release medium and dissolved oxygen probe. Dissolved oxygen levels were monitored and recorded over time using the HI5421 probe. From the resulting dissolved oxygen-time profiles, a maximum concentration (C_max_) was extracted for each sample and used as a measure of oxygen loading and release. Statistical comparisons were conducted by performing unpaired two-tailed t-tests of dissolved oxygen measurements at each time. All experiments were done in triplicate on samples less than 1 week after preparation.

### ^19^F Nuclear magnetic resonance characterization

Nuclear magnetic resonance (NMR) spectroscopy was used qualitatively in all PFC nanoemulsions and to measure the concentration of ^19^F nuclei in representative nanoemulsions. ^19^F NMR chemical shifts were reported as ppm using a 0.4% (v/v) trifluoroacetic acid (TFA) solution as internal reference with chemical shift set at − 76.0 ppm. PCE nanoemulsions showed a singlet peak at − 92.4 ppm and PFOB nanoemulsions showed a triplet peak at − 83.7 ppm chosen for quantitation. The resulting spectral peaks were integrated, and the resulting areas were used to calculate the mean ^19^F concentration (n = 1 per NE). Spectra were obtained on a Bruker Avance III at 500 MHz. Samples were prepared by adding 200 μL emulsion, 200 μL 0.4% w/v trifluoroacetic acid (TFA) as an internal standard, and 50 μL deuterium oxide in a NMR sample tube (7″ length, 4.1 mm inner diameter) (Wilmad-LabGlass, Vineland, NJ, USA). PFC loading was calculated as purity of analyte (*P*_*ana*_):

$$P_{ana} = \frac{{I_{ana} }}{{I_{Std} }} \times \frac{{N_{Std} }}{{N_{ana} }} \times \frac{{M_{ana} }}{{M_{Std} }} \times \frac{{m_{Std} }}{{m_{Sample} }} \times P_{Std}$$

where *I*_*ana*_, *N*_*ana*_, *M*_*ana*_, are the NMR peak integral, number of nuclei, and molecular weight for the analyte (perfluorocarbon), and *I*_*Std*_, *N*_*Std*_, *M*_*Std*_, and *P*_*Std*_ are the NMR peak integral, number of nuclei, molecular weight, and purity of the internal standard^56^. NMR spectra are shown in Supplemental Figures S7-S15.

### Software information

NMR spectra were analyzed and plotted in Bruker TopSpin 4.0.6 (https://www.bruker.com/en/products-and-solutions/mr/nmr-software/topspin.html). Dissolved oxygen data and PFC loading data were analyzed and plotted in GraphPad Prism 8.4.3 (https://www.graphpad.com/scientific-software/prism/). FMECA summary was plotted in JMP Pro 14.3.0 (https://www.jmp.com/en_us/software/predictive-analytics-software.html). MLR analysis and graphing was completed in JMP Pro 14.3.0. Graphics depicting NE droplet configuration, oxygen release experimental setup, and oxygen flow mechanics were created in Microsoft PowerPoint for Microsoft 365, version 16.0.13127.21062 (https://www.microsoft.com/en-us/microsoft-365/powerpoint) and ChemDraw Professional 19.1.0.8 (https://www.perkinelmer.com/category/chemdraw). Cluster analysis scatterplot of NE size and PDI was created in JMP Pro 14.3.0. All other DLS data were analyzed and graphed in GraphPad Prism 8.4.3. Balance schematic was created in ChemDraw.

## Supplementary Information

Supplementary information 1.

Supplementary information 2.

Supplementary information 3.

## Data Availability

Document named *Raw DO compiled and controls_Fileshare* contains raw dissolved oxygen measurements from in vitro oxygen release from all design points and controls. Each measurement is average of three independent experiments. Document named *FMECA_O2-NE* contains the unabridged failure mode, effects, and criticality analysis with risk priority number rankings.
