# Supplementary material for: Quality by design approach identifies critical parameters driving oxygen delivery performance in vitro for perfluorocarbon based artificial oxygen carriers
Source: Sci Rep. 2021 Mar 10;11:5569. doi: 10.1038/s41598-021-84076-1 (PMC7946885; doi:10.1038/s41598-021-84076-1)
Supplement: Supplementary file 1 — Supplementary information 1. [file 41598_2021_84076_MOESM1_ESM.pdf]

Supporting information

Quality by Design Approach Identifies Critical Parameters Driving Oxygen Delivery Performance in Vitro for Perfluorocarbon Based Artificial Oxygen Carriers

*Eric Lambert and Jelena M. Janjic**

Graduate School of Pharmaceutical Sciences, Duquesne University, 600 Forbes Avenue, Pittsburgh, PA 15282, USA.

*Correspondence: janjicj@duq.edu

Supplemental Figure S1. .... *2*
Supplemental Figure S2. .... *3*
Supplemental Figure S3. .... *4*
Supplemental Figure S4.. .... *5*
Supplemental Figure S5. .... *6*
Supplemental Figure S6. .... *7*
Supplemental Figure S7. .... *10*
Supplemental Figure S8. .... *11*
Supplemental Figure S9. .... *12*
Supplemental Figure S10. .... *13*
Supplemental Figure S11. .... *14*
Supplemental Figure S12. .... *15*
Supplemental Figure S13. .... *16*
Supplemental Figure S14. .... *17*
Supplemental Figure S15.. .... *18*

Supplemental Table S1. .... *8*
Supplemental Table S2. .... *9*

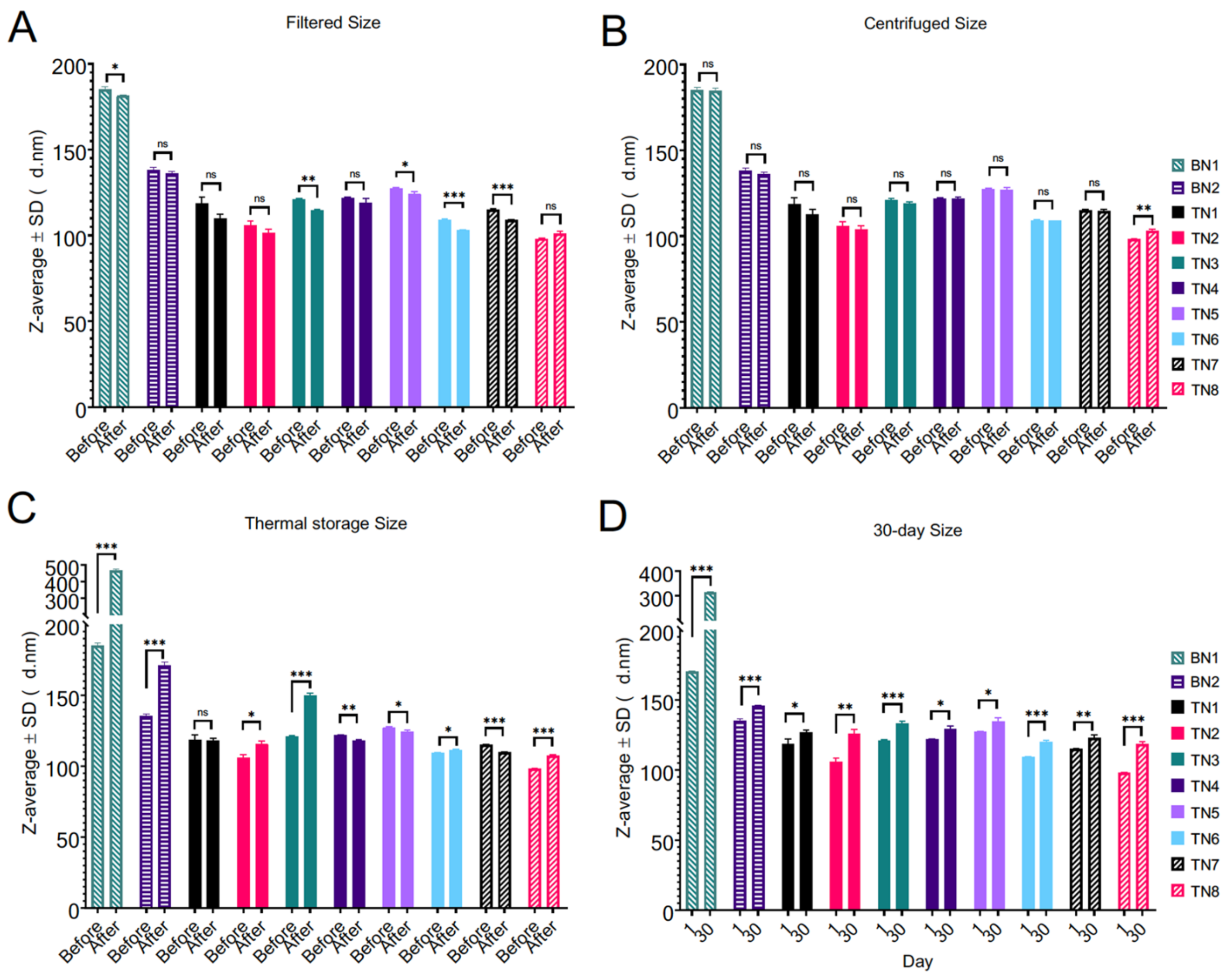

Supplemental Figure S1. Z-average diameter evaluation of biphasic and triphasic nanoemulsions (BN and TN, respectively). (A) Stability to filtration through 0.22 um syringe filter was good in all samples. (B) Stability in centrifugation conditions (16.1×*g* for 5 minutes) was good in all samples. (C) Storage in 50 °C for 2 weeks resulted in destabilization of biphasic emulsions. (D) 30-day storage at 4°C shows a 30-day z-average measurement compared to a 1-day-old measurement for comparison. 4°C storage resulted in destabilization of PFOB biphasic formulation only (BN1). All measurements are mean ± standard deviation from 3 measurements. ns, *, **, *** indicate not significant, $p>0.05$, $p<0.05$, $p<0.01$, and $p<0.001$, respectively.

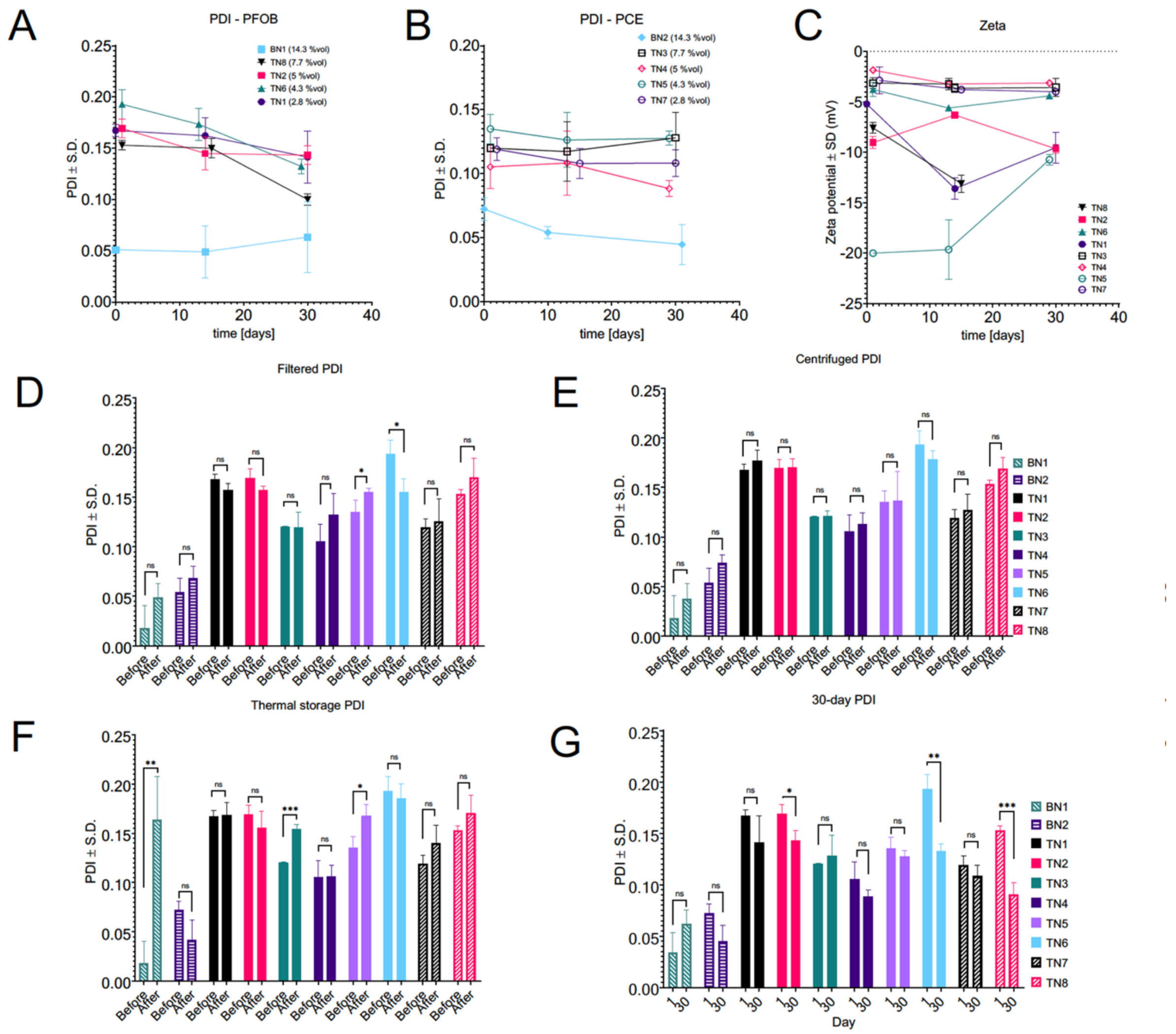

Supplemental Figure S2. Size distribution (PDI) and zeta potential evaluation of biphasic and triphasic nanoemulsions (BN and TN, respectively). (A, B) Nanoemulsion PDI was followed for 30 days to evaluate stability in refrigerated storage conditions (4 °C). (C) Nanoemulsion zeta potential was followed for 30 days to evaluate stability in refrigerated storage conditions (4 °C). No evidence of destabilization occurs from observing PDI and zeta potential. (D) Stability to filtration through 0.22 um syringe filter was good in all samples. (E) Stability in centrifugation conditions (16.1×*g* for 5 minutes) was good in all samples. (F) Storage in 50 °C for 2 weeks resulted in destabilization of biphasic PFOB emulsion. (G) 30-day storage at 4°C shows a 30-day z-average measurement compared to a 1-day-old measurement for comparison. 30-day storage at 4°C was acceptable in all emulsions. All measurements are mean ± standard deviation from 3 measurements. ns, *, **, *** indicate not significant, $p>0.05$, $p<0.05$, $p<0.01$, and $p<0.001$, respectively. %vol concentration numbers indicate the concentration of perfluorocarbon.

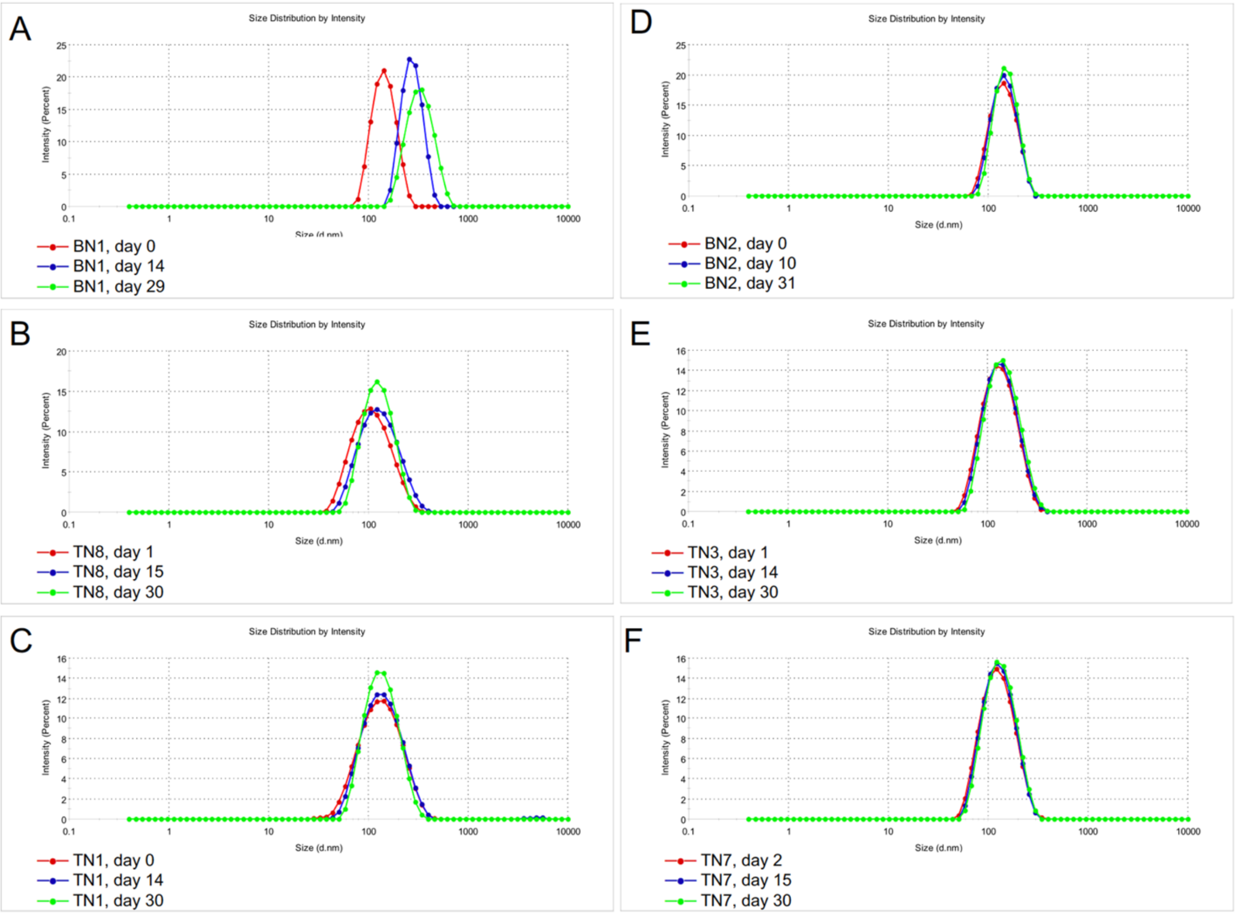

Supplemental Figure S3. Size distribution overlays of representative perfluorocarbon nanoemulsions (PFC-NEs). Each panel overlays one representative NE at 3 different ages, up to 31 days after production, as indicated in the figure legends. Selected PFC-NEs are biphasic perfluorooctyl bromide (PFOB) and perfluoro-15-crown-5-ether (PCE) NEs, and triphasic NEs comprised of highest and lowest PFC concentration. PFOB NEs appear in A, B, and C, while PCE NEs appear in D, E, and F. These comparisons show that the size distribution of BN1 shifts to higher sizes rapidly, while BN2 and TNs approximately maintain constant distribution. BN, biphasic nanoemulsion; TN, triphasic nanoemulsion.

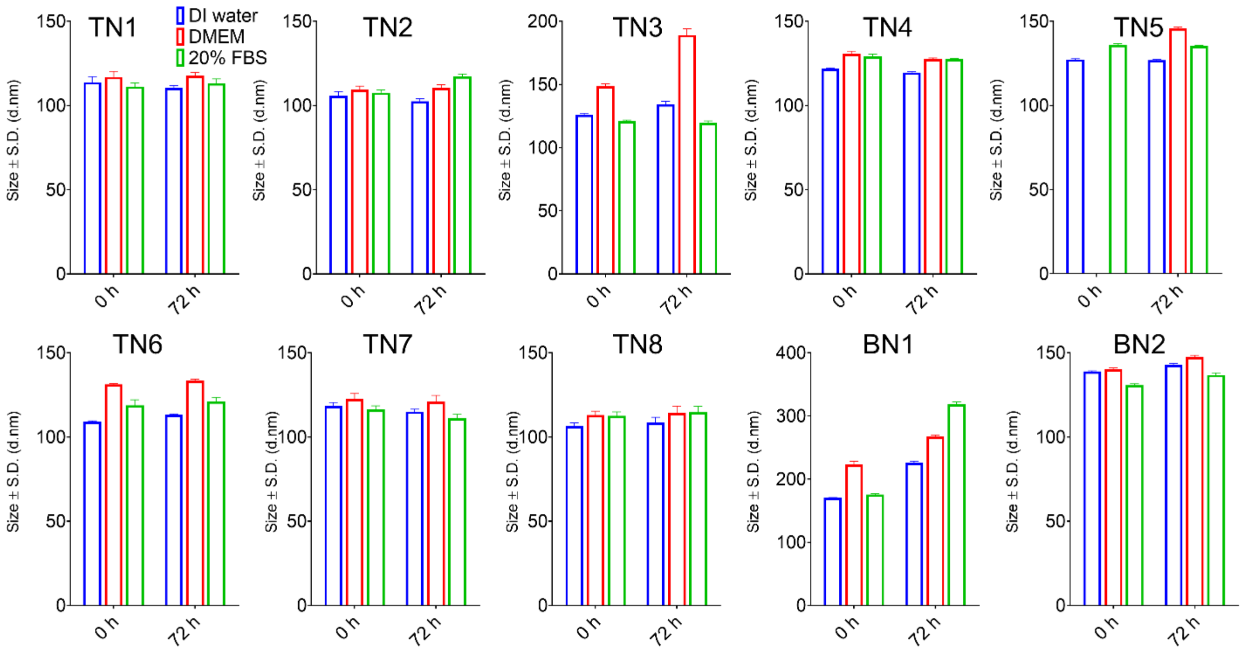

Supplemental Figure S4. Graphs of z-average size in response to serum stability test. Samples were incubated for 72 hours in biological media at 37 °C and size was recorded before and after. All measurements are mean ± standard deviation from 3 measurements. TN, triphasic nanoemulsion; BN, biphasic nanoemulsion; DI, deionized; DMEM, Dulbecco's Modified Eagle's Medium; FBS, fetal bovine serum.

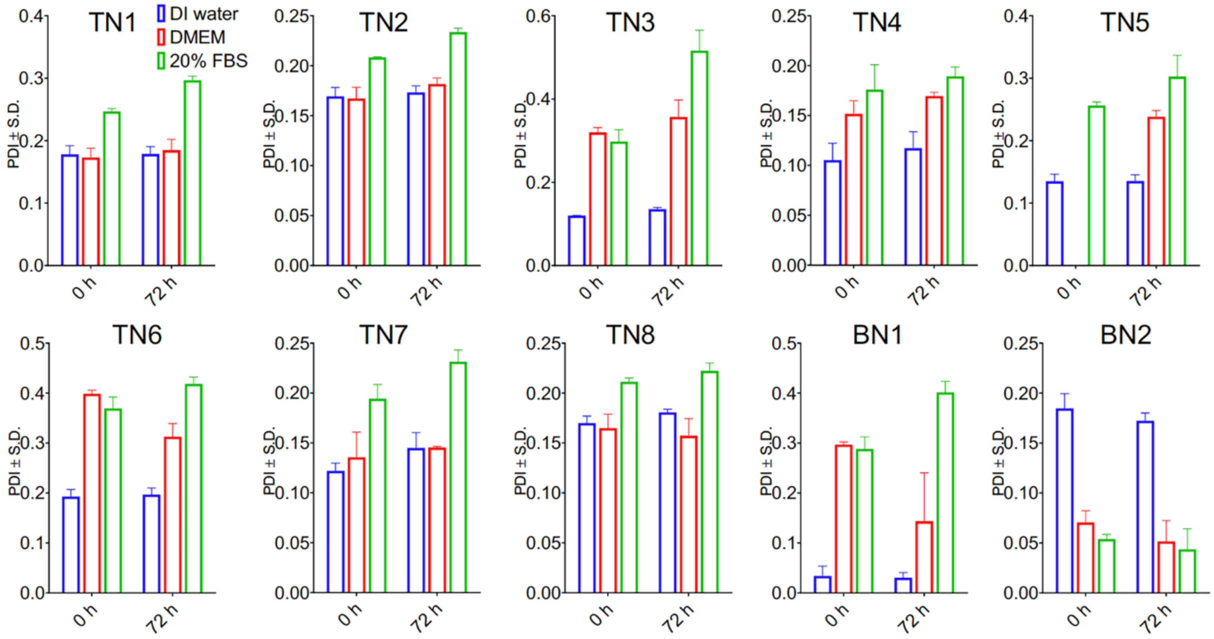

Supplemental Figure S5. Graphs of polydispersity index (PDI) in response to serum stability test. Samples were incubated for 72 hours in biological media at 37 °C and PDI was recorded before and after. All measurements are mean ± standard deviation from 3 measurements. DI, deionized; DMEM, Dulbecco's Modified Eagle's Medium; FBS, fetal bovine serum.

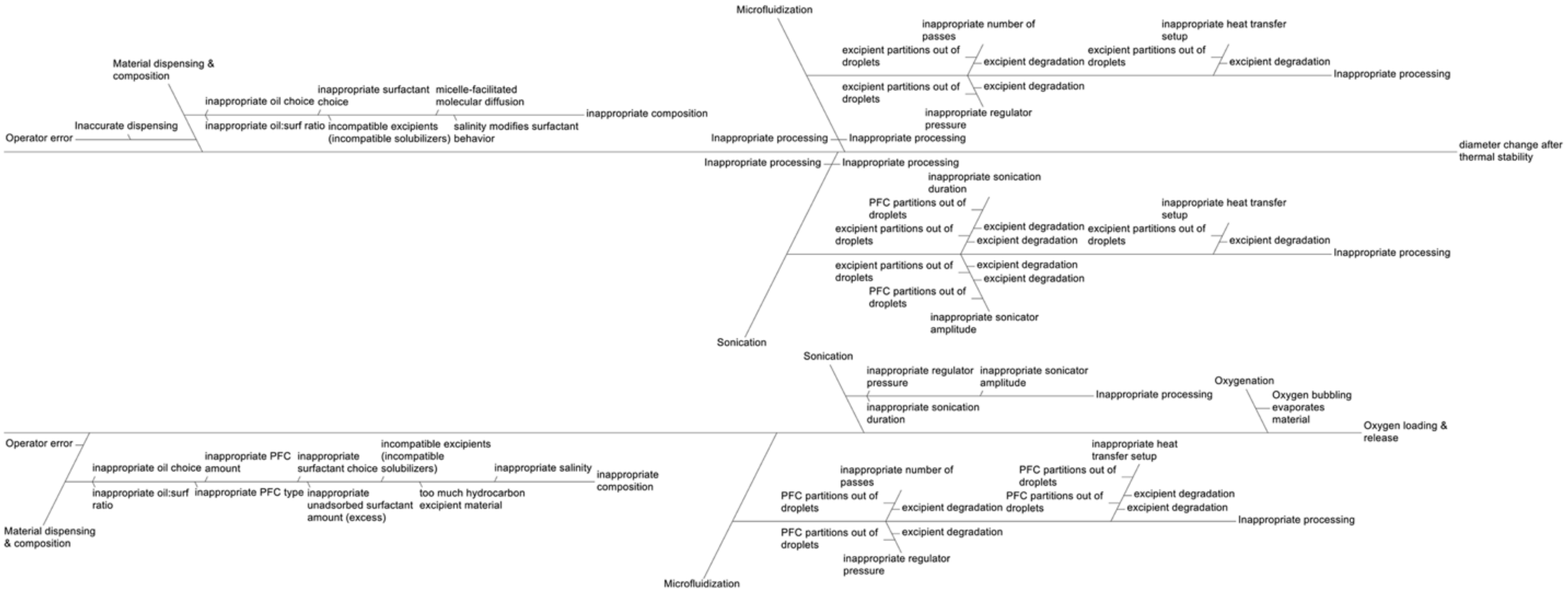

Supplemental Figure S6. Cause and effect diagrams for oxygen loading & release and size change after thermal stability. All branches of the diagram represent sources of variability of the quality attribute in question.

Supplemental Table S1. Multiple linear regression (MLR) was used to fit models to two attributes (responses): oxygen release C_{max} and thermal stability % size change. These two responses make up the backbone of a successful artificial oxygen carrier. Goodness of fit statistics for reduced models is demonstrated by R^2, root mean square error (RMSE), and leave-one-out cross validation (LOOCV) RMSE.

	C_{max}	Thermal stability Δdiameter
Mean of Response	1.462 (mg/L)	4.25 (%)
Number of samples	8	8
R^2	0.9950	0.9708
Adjusted R^2	0.9883	0.9489
Root Mean Square Error	0.027 (mg/L)	2.179 (%)
LOOCV RMSE	0.065 (mg/L)	2.777 (%)

Supplemental Table S2. Analysis of variance (ANOVA) for reduced MLR models. ANOVA compares the variance explained by the model and the variance unaccounted for (error) and based off this calculates the F ratio. From the F ratio at the specified degrees of freedom (DF), a p-value (Prob > F) is calculated for each model. Significance was taken at $p < 0.05$.

	C_{max} (mg/L)					Thermal stability Δsize (%)				
Source	DF	Sum of Squares	Mean Square	F Ratio	Prob > F	DF	Sum of Squares	Mean Square	F Ratio	Prob > F
Model	4	0.433659	0.108415	148.7484	0.0009	3	631.405	210.468	44.321	0.0016
Error	3	0.002187	0.000729			4	18.995	4.749		
Corrected Total	7	0.435846				7	650.400			

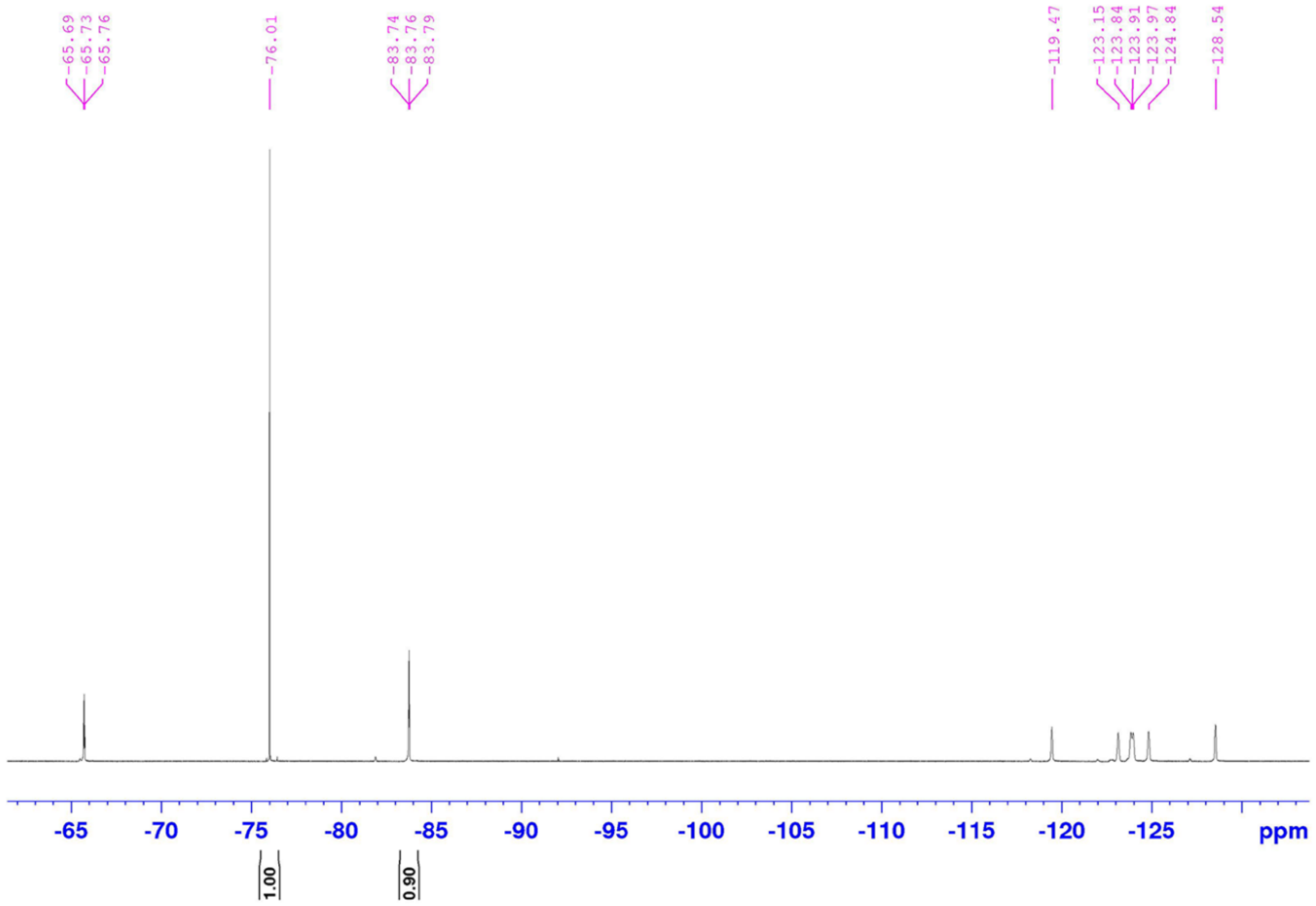

Supplemental Figure S7. ^{19}F NMR spectra of TN1. Reference compound trifluoroacetic acid displays chemical shift at -7

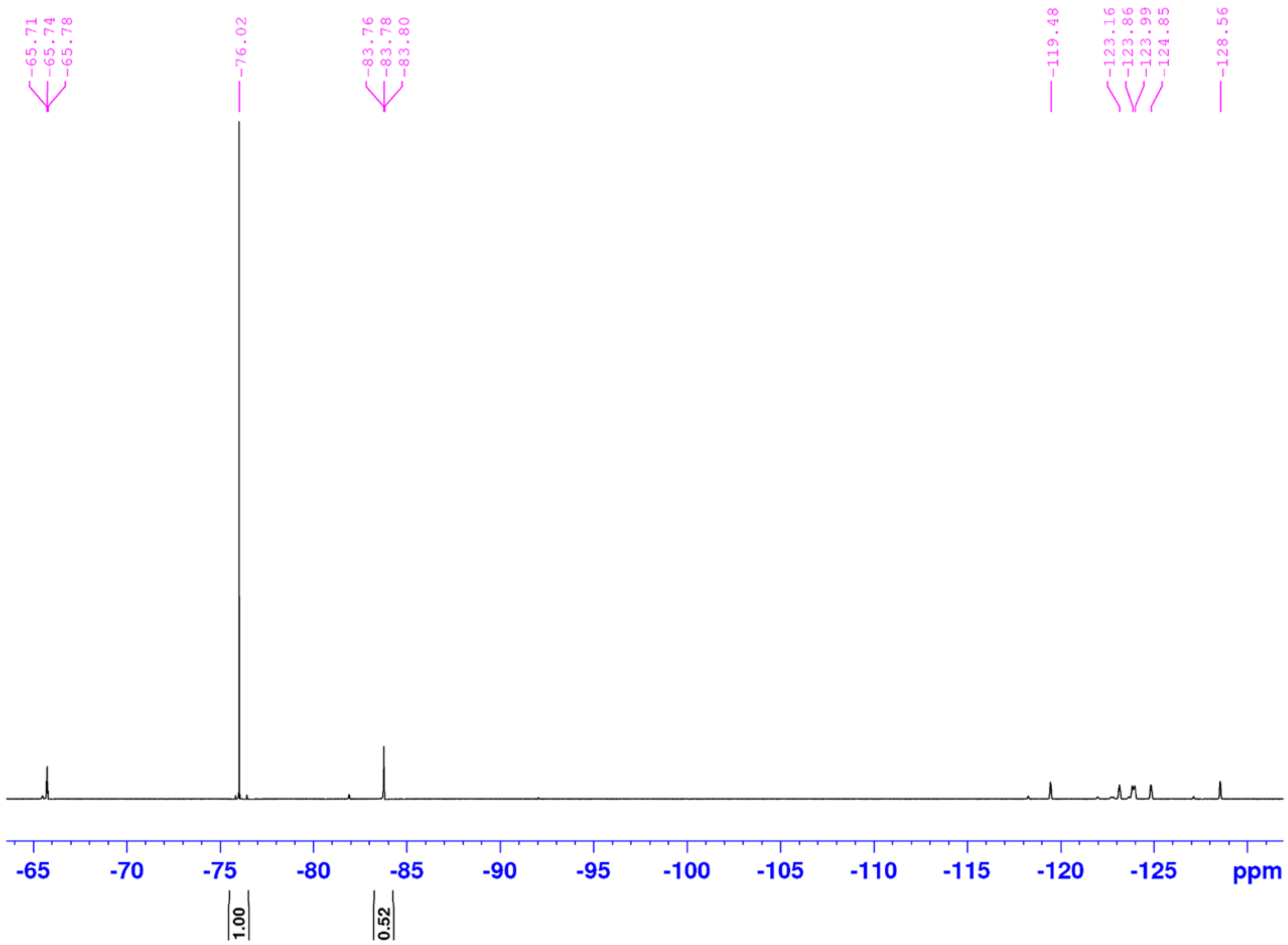

Supplemental Figure S8. ^{19}F NMR spectra of TN2. Reference compound trifluoroacetic acid displays chemical shift at -76 ppm.

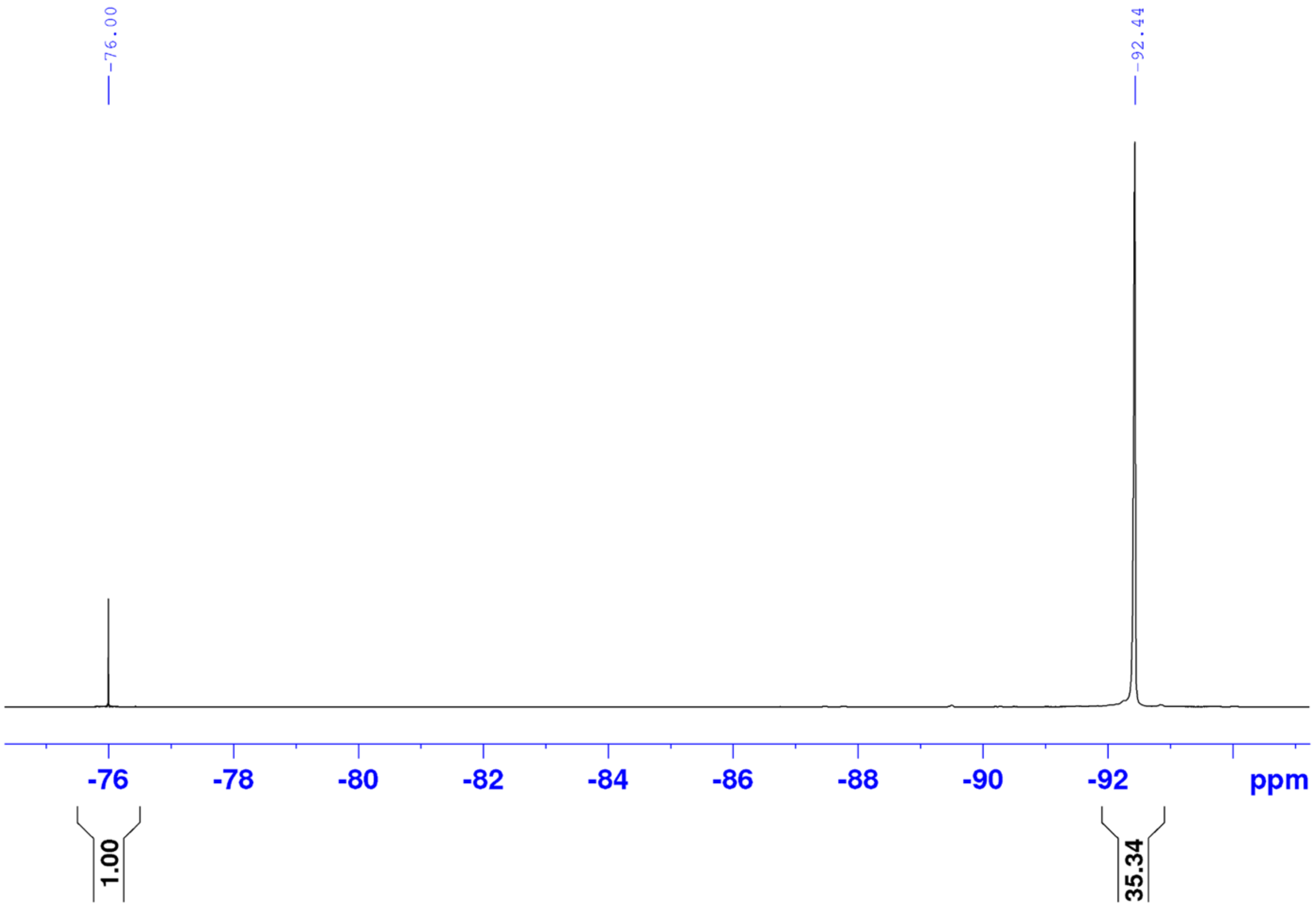

Supplemental Figure S9. ^{19}F NMR spectra of TN3. Reference compound trifluoroacetic acid displays chemical shift at -76 ppm.

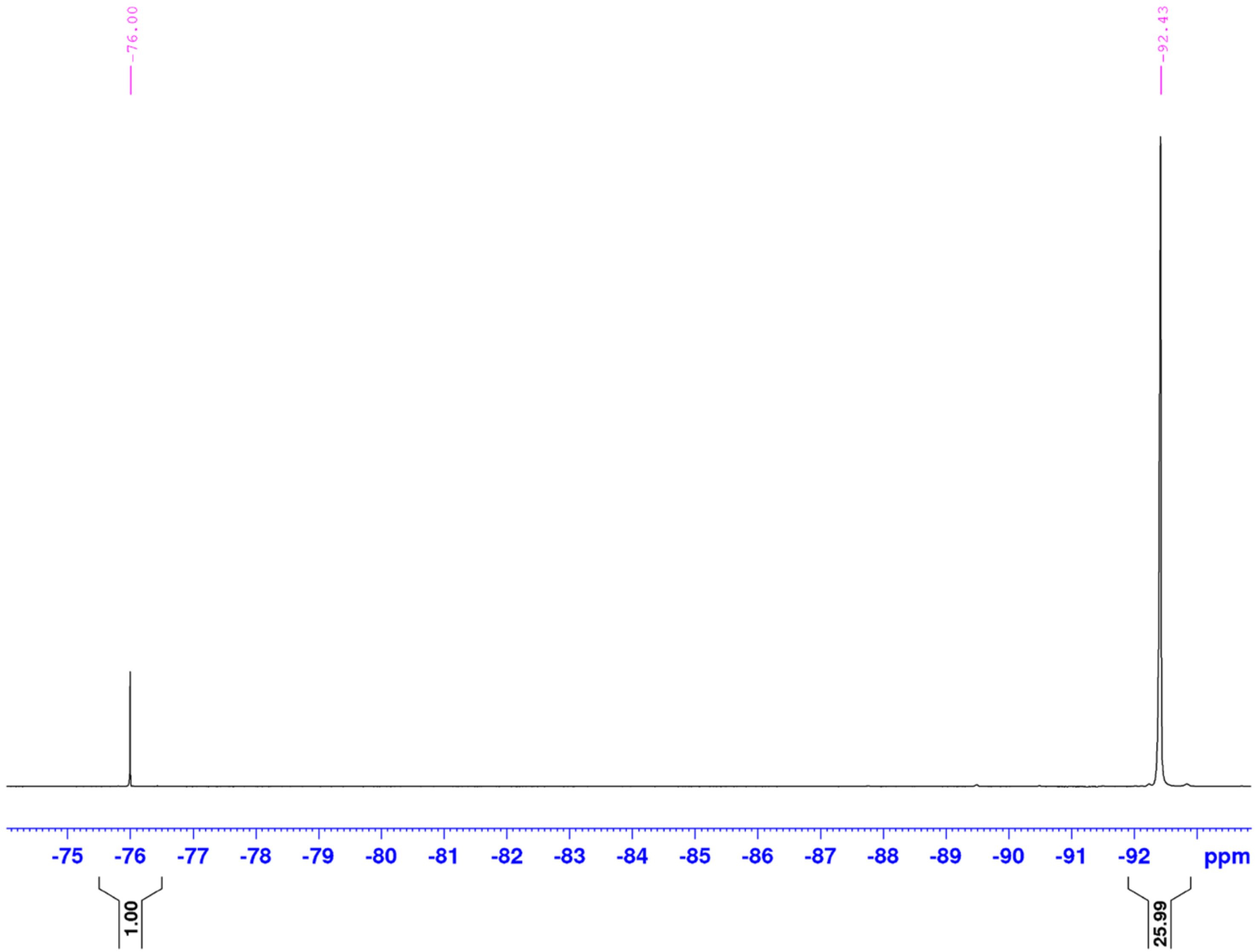

Supplemental Figure S10. ^{19}F NMR spectra of TN4. Reference compound trifluoroacetic acid displays chemical shift at -76 ppm.

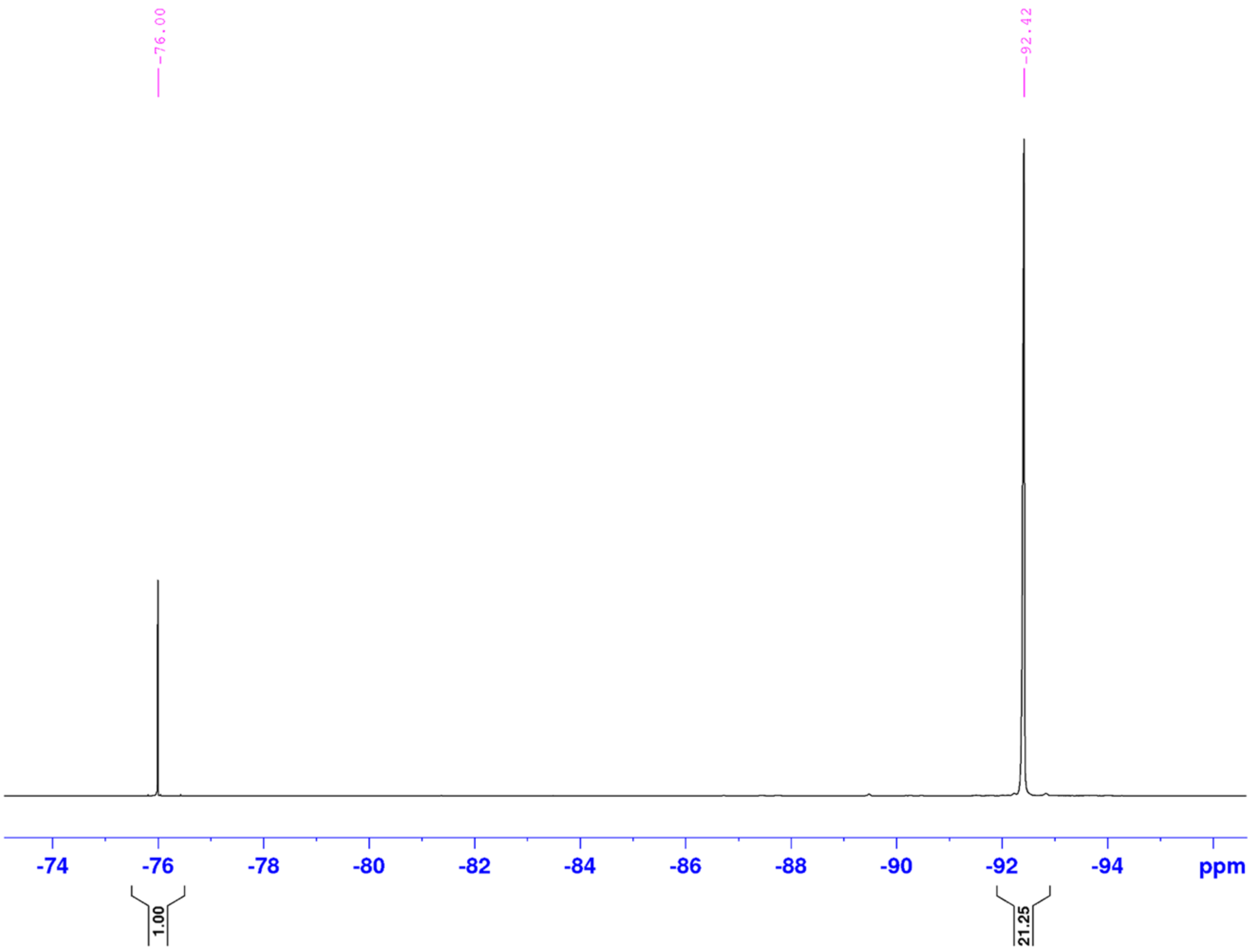

Supplemental Figure S11. ^{19}F NMR spectra of TN5. Reference compound trifluoroacetic acid displays chemical shift at -76 ppm.

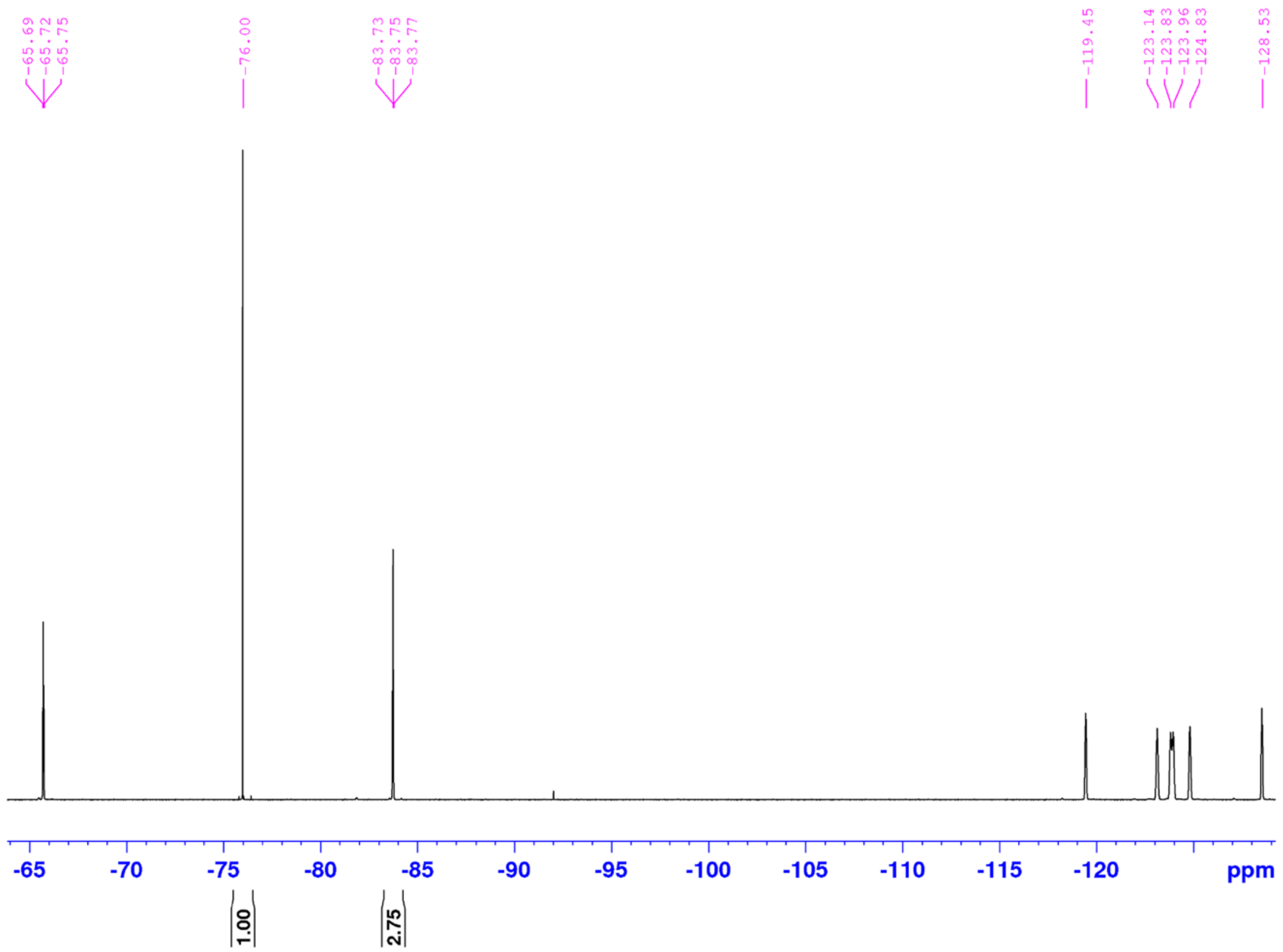

Supplemental Figure S12. ^{19}F NMR spectra of TN6. Reference compound trifluoroacetic acid displays chemical shift at -76 ppm.

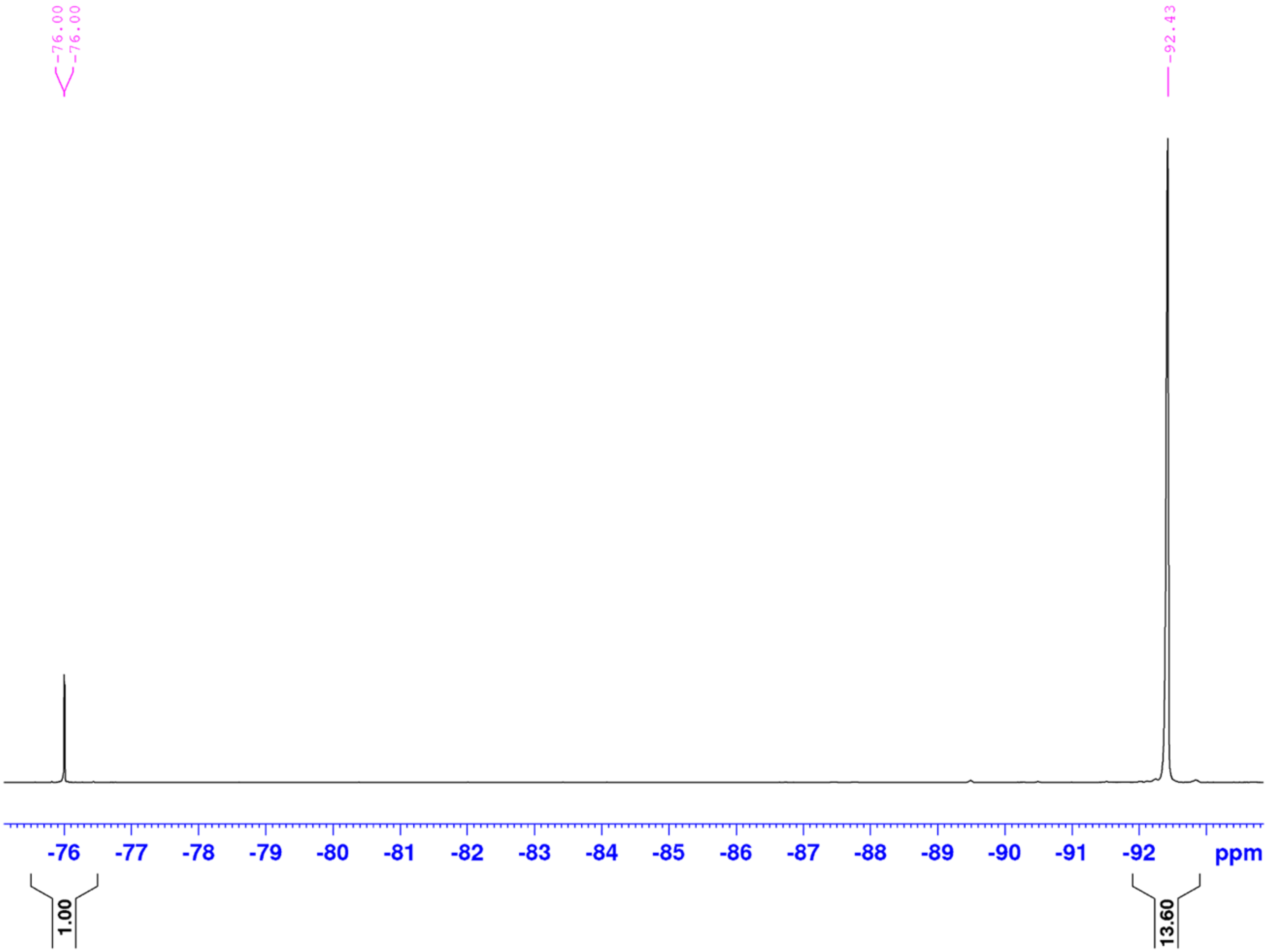

Supplemental Figure S13. ^{19}F NMR spectra of TN7. Reference compound trifluoroacetic acid displays chemical shift at -76 ppm.

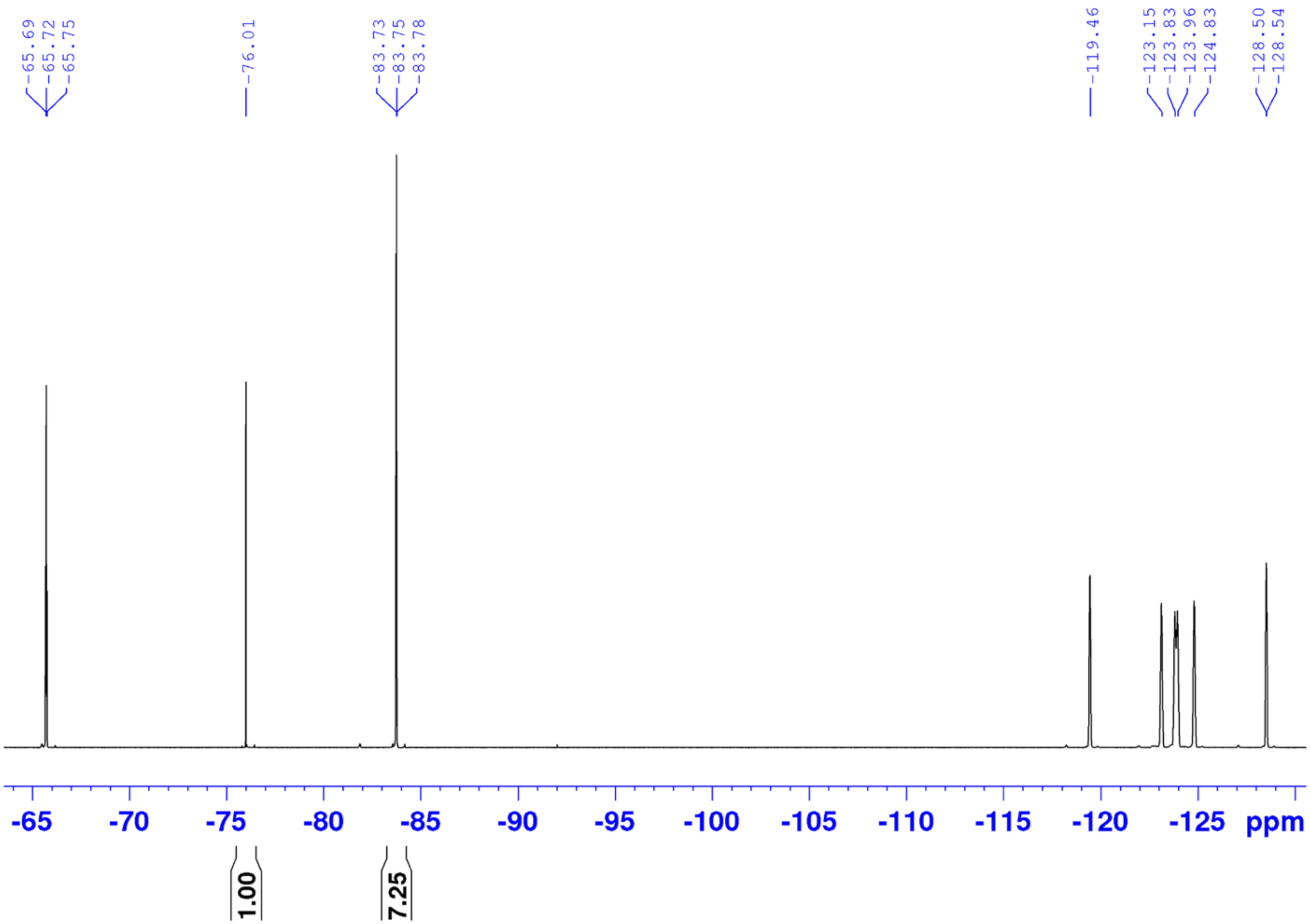

Supplemental Figure S14. ^{19}F NMR spectra of TN8. Reference compound trifluoroacetic acid displays chemical shift at -76 ppm.

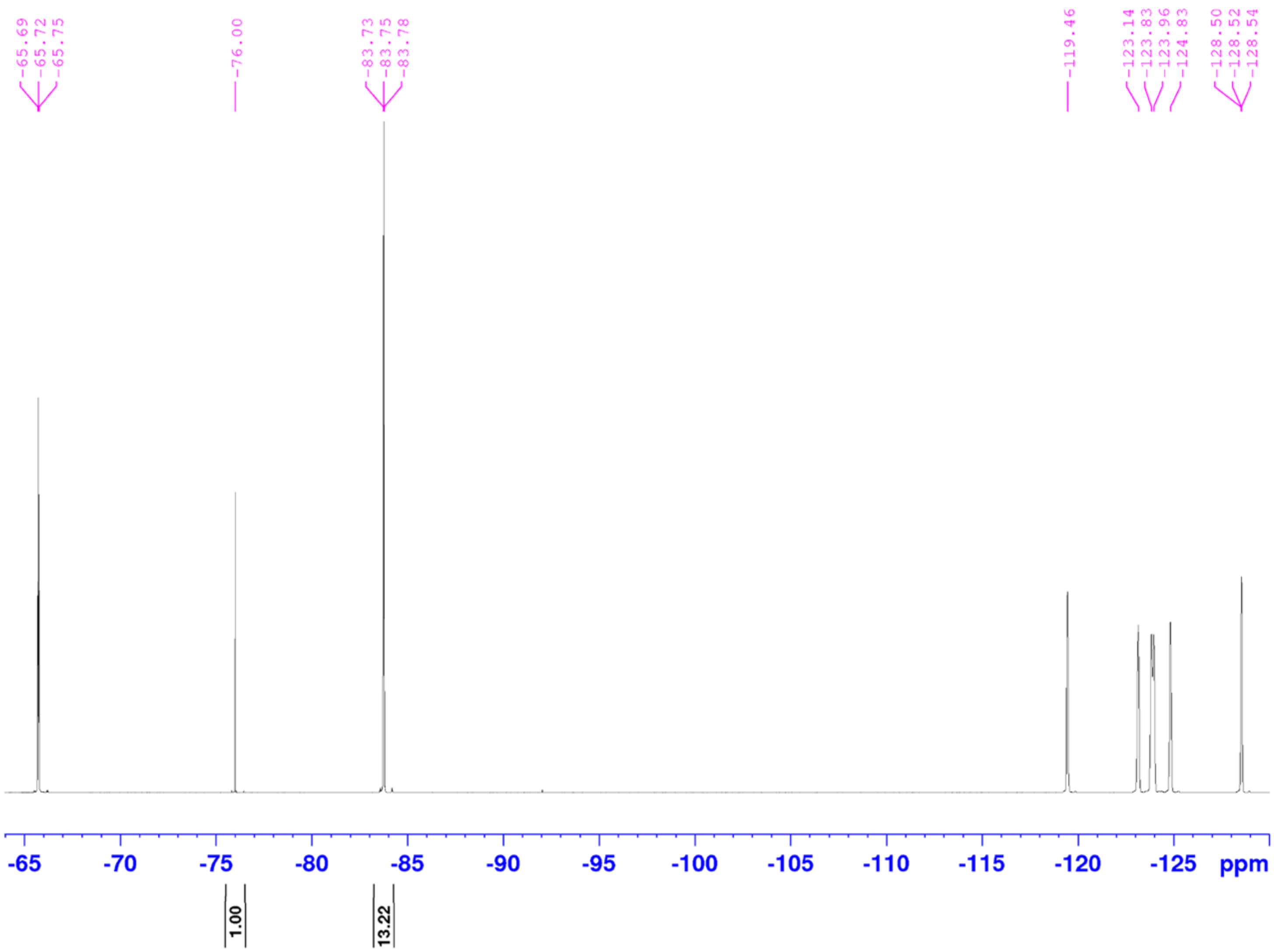

Supplemental Figure S15. ^{19}F NMR spectra of BN1. Reference compound trifluoroacetic acid displays chemical shift at -76 ppm.
